# [^18^F]RO948 Tau PET in early and late onset Alzheimer’s disease: associations with plasma p-tau 217, atrophy and cognition

**DOI:** 10.1007/s00259-026-08015-w

**Published:** 2026-06-26

**Authors:** Mariola Zapater-Fajari, Marco Bucci, Konstantinos Chiotis, Ove Almkvist, Anders Wall, Jonas Eriksson, Gunnar Antoni, Ilaria Pola, Kübra Tan, Wiebke Traichel, Andrea L. Benedet, Nicholas J. Ashton, Henrik Zetterberg, Nenad Bogdanovic, Agneta Nordberg

**Affiliations:** 1 Department of Neurobiology, Care Sciences and Society, Centre for Alzheimer Research, Division of Clinical Geriatrics, Karolinska Institutet, Neo 7th Floor, Stockholm, 14183 Sweden; 2 Turku PET Center, Turku University Central Hospital, University of Turku and Åbo Akademi University, Turku, 20251 Finland; 3 Theme Inflammation and Aging, Karolinska University Hospital, Stockholm, 14186 Sweden; 4 Department of Surgical Sciences, Section of Molecular Imaging & Medical Physics, Uppsala University, Uppsala, 75185 Sweden; 5 PET Centre, Uppsala University Hospital, Uppsala, 75185 Sweden; 6 Department of Medicinal Chemistry, Uppsala University, Uppsala, 75123 Sweden; 7 Department of Psychiatry and Neurochemistry, Institute of Neuroscience and Physiology, The Sahlgrenska Academy, University of Gothenburg, 413 45, Gothenburg, Sweden; 8 Section of Forensic Chemistry, Department of Forensic Medicine, Faculty of Health Sciences, University of Copenhagen, Frederik V’s Vej 11, 3, Copenhagen, 2100 Denmark; 9 Institute of Psychiatry, Psychology & Neuroscience, King’s College London, Maurice Wohl Clinical Neuroscience Institute, London, SE5 9RT UK; 10 NIHR Biomedical Research Centre for Mental Health & Biomedical Research Unit for Dementia at South London & Maudsley NHS Foundation, London, SE5 8AF UK; 11 Clinical Neurochemistry Laboratory, Sahlgrenska University Hospital, Mölndal, 43180 Sweden; 12 Department of Neurodegenerative Disease, UCL Institute of Neurology, Queen Square, London, WC1N 3BG UK; 13 Hong Kong Center for Neurodegenerative Diseases, Clear Water Bay, Hong Kong, 999077 China; 14 Wisconsin Alzheimer’s Disease Research Center, School of Medicine and Public Health, University of Wisconsin-Madison, Madison, 53792-2420 USA; 15 Department of Pathology and Laboratory Medicine, School of Medicine and Public Health, University of Wisconsin-Madison, Madison, 53792-2420 USA; 16 Centre for Brain Research, Indian Institute of Science, C.V. Raman Avenue, Bangalore, 560 012 India

**Keywords:** Positron emission tomography (PET), Alzheimer’s disease biomarkers, Neurofibrillary tau tangles, Blood-based biomarkers, Cognition, Neurodegeneration

## Abstract

**Purpose:**

The aim of this study was to compare regional brain tau extent across the disease spectrum in early- and late-onset Alzheimer’s disease using the second-generation tau PET tracer [^18^F]RO948, and to investigate the relationship between PET-derived patterns, plasma p-tau217, brain atrophy, and cognition.

**Methods:**

We examined 57 participants: 39 patients from the Cognitive Assessment Unit at Karolinska University Hospital (Stockholm/Sweden)—diagnosed as MCI Aβ– (*n* = 10), MCI Aβ + (*n* = 16; 8 EO and 6 LO), and AD (*n* = 13; 5 EOAD and 8 LOAD)—and 18 cognitively normal controls. All participants underwent [^18^F]RO948 tau-PET, structural MRI, cognitive testing, and plasma p-tau217 analysis.

**Results:**

Both EO and LO MCI (Aß +) showed higher [^18^F]RO948 binding in amygdala, entorhinal cortex, hippocampus (*p* < 0.001) and extending to inferior temporal regions (*p* < 0.01) compared to controls, with some MCI-EO cases already showing advanced neocortical tau burden. At the AD stage, EOAD patients showed greater neocortical tau extent in temporo-parietal and frontal cortices than LOAD. P‑tau217 positively correlated with [^18^F]RO948 in amygdala, entorhinal cortex, hippocampus (rho = 0.81) followed by temporal (rho = 0.71), and parietal cortices (rho = 0.56, all *p* < 0.001). The Tau‑PET/p-tau217 association was different between EOAD and LOAD with greater differences in parietal cortices. Tau‑PET also showed stronger region‑specific associations with cognitive impairment than p‑tau217, especially in EOAD.

**Conclusions:**

[^18^F]RO948-PET captured interindividual heterogeneity in tau accumulation that was not reflected by plasma p-tau217. This dissociation was particularly evident in neocortical regions, where LOAD showed elevated plasma p-tau217 levels despite low tau PET binding. These findings highlight the value of tau-PET as a sensitive biomarker for detecting early disease heterogeneity, assessing disease severity, and improving patient stratification for disease-modifying treatments.

**Supplementary Information:**

The online version contains supplementary material available at https://doi.org/10.1007/s00259-026-08015-w.

## Introduction

Alzheimer's disease (AD) is a progressive neurodegenerative disorder characterized by multiple pathological events occurring during the decade(s) of disease progression. Accumulation of amyloid-β (Aβ) plaques and hyperphosphorylated tau neurofibrillary tangles have been considered key pathological markers of AD but recent data underline the importance of early changes in fluid biomarkers [1]. Gradual accumulation of amyloid-β (Aβ) in the brain is widely considered the initiating event in the AD pathological cascade, acting as a driving factor that facilitates tau pathology, which in turn shows stronger associations with neurodegeneration and clinical symptoms [2]. Tau pathology follows a stereotypical anatomical trajectory, beginning in the transentorhinal and subcortical regions and subsequently spreading to associative neocortical areas, a pattern classically described by Braak staging [3, 4]. The development of tau positron emission tomography (PET) imaging has enabled the in vivo visualization of this progression, closely mirroring clinical symptoms, providing insights into disease staging and prognosis [5], and predicting subsequent brain atrophy [6–9]. However, considerable heterogeneity exists in the regional distribution and progression of tau pathology across individuals. Factors such as age at disease onset, genetic background, and sex contribute to these differences and are thought to underlie distinct clinical phenotypes of AD [10, 11]. Most current knowledge regarding tau spatial extent and its heterogeneity derives from studies using the first-generation tracer [^18^F]Flortaucipir ([^18^F]AV-1451) [2, 12], which is affected by off-target binding in important regions like the choroid plexus (particularly compromising evaluation of tracer uptake in the medial temporal lobe, one of the earliest regions to show tau pathology) [13, 14]. Second-generation tau-PET tracers offer improved specificity and lower non-specific binding, with [^18^F]MK6240 detecting earlier tau accumulation than Flortaucipir [15], and [^18^F]RO948 better differentiating AD from other neurodegenerative dementias [16]. Despite these advantages, no study to date has comprehensively characterized the full regional extent of tau using [^18^F]RO948 across the AD spectrum, including subcortical structures that are critical for early Braak stages.

The present study aimed to provide a deeper understanding of the regional brain extent of tau across the AD spectrum using the second-generation tau-PET tracer [^18^F]RO948, considering age-at onset subtypes and explore its relationships with plasma p-tau217. Plasma p-tau217 has been demonstrated as a reliable and scalable biomarker for AD, offering potential utility for early detection and disease monitoring [17, 18]. However, before plasma biomarkers can be implemented in clinical settings, it is of highly relevance and importance to investigate their relationship with the more established imaging and CSF biomarkers of AD. Furthermore, the extent to which tau-PET and plasma p-tau217 differentially relate to cognition and cortical atrophy by age-at onset subtypes remains unexplored. Integrating tau-PET and MRI imaging with fluid biomarkers in a clinical cohort offers a promising approach to better understand the biological heterogeneity of AD and may shed light on more personalized diagnostic and therapeutic strategies [19].

## Materials and methods

### Study design and participants

This study consisted of 57 participants of which 39 were recruited from the Cognitive Assessment Unit at Karolinska University Hospital Huddinge, Stockholm, Sweden, to which they initially had been referred for assessments due to cognitive problems and 18 cognitively normal individuals (10 recruited from the Clinical Trial Consultants AB (Uppsala, Sweden), and 8 participant (non-carriers) from a longitudinal observational study of familial autosomal dominant AD at Karolinska Institutet. None of the MCI or AD patients participating in the study was known to carry any autosomal dominant mutation (PSEN1, APP). At the Cognitive Assessment Unit, patients underwent an extensive clinical evaluation including comprehensive history, physical and neurological exam, cognitive assessment, CSF sampling for both total tau (CSF t-tau), phosphorylated tau (CSF p-tau181), and amyloid-beta (CSF Aβ40 and Aβ42), CT or MRI, and for some cases [^18^F]FDG-PET and amyloid-PET ([^18^F]flutemetamol or [^11^C]PIB). Consensus diagnosis was established by a multidisciplinary dementia expert team comprising specialists in cognitive disorders, clinical neuropsychologists, and specialist nurses. The patients were diagnosed as Mild Cognitive Impairment (MCI) Aβ-, MCI Aβ + and AD. Furthermore, MCI Aβ + and AD groups were further diagnosed according to age at-onset of the disease (65 years cut-off) as MCI Aβ + Early-Onset (MCI-EO) and MCI Aβ + Late-Onset (MCI-LO), and AD Early-Onset (EOAD) and AD Late-Onset (LOAD). During the experimental day, all 57 participants underwent, brain MRI, tau-PET [^18^F]RO948 and plasma sampling at the PET Center at Uppsala University (Uppsala, Sweden).

### PET and MRI Image acquisition and processing

Dynamic [^18^F]RO948 PET scans were acquired 70–90 min post-injection (3 MBq/kg; 150–300 MBq) on a GE Discovery MI PET/CT system and reconstructed using the VPFX-S algorithm. The 3D T1-weighted magnetic resonance imaging (MRI) scans were acquired in a SIGNA PET/MR 3.0 T MRI scanner (General Electric Medical Systems, USA), using a 3D inversion recovery fast spoiled gradient echo sequence (IR-FSPGR; GE BRAVO equivalent) with 1-mm isotropic voxel resolution (TR = 8.49, TE = 3.25, TI = 450 ms, flip = 12**°)**. Three patients were scanned at Karolinska University Hospital, Huddinge due to having a pacemaker, using comparable 1.5 T or 3 T clinical MRI systems. For one participant without MRI, a CT-derived synthetic MPRAGE image was generated using SynthSR [20].

The resulting PET images were motion corrected, summed and co-registered to their corresponding T1-weighted MR images using SPM12 (Wellcome Department of Cognitive Neurology, London, UK; http://www.fil.ion.ucl.ac.uk/spm) and an in-house adaptation of the medical imaging pipeline MAGIA (https://aivo.utu.fi/magia/) [21]. Standardized uptake value ratio (SUVR) images were generated using the inferior cerebellar cortex as reference region (SUIT atlas) [13, 22, 23]. All tau PET images were visually inspected for potential off-target binding. Mild off-target signal was observed in a small number of participants, primarily in regions previously reported for [^18^F]RO948 [13], including meninges and white matter (Supplementary Fig. 1).

Cortical and subcortical segmentations were obtained using FreeSurfer (v7.4.1) based on the Desikan–Killiany atlas [24]. Mean regional SUVR values were extracted from selected regions of interest (ROIs) previously implicated in early tau deposition [2, 25] and age-at-onset–related differences [26, 27], including medial temporal and lateral temporal regions, as well as frontal, parietal, and occipital composite ROIs. All ROIs were averaged across the hemispheres. Voxelwise SUVR maps were normalized to MNI152 space. Cortical thickness was calculated within FreeSurfer-derived ROIs [28]. Hippocampal and amygdala volumes were extracted from the Freesurfer automated volumetric segmentation and divided by the estimated Total Intracranial Volume (TIV) [29].

### Plasma p-tau217

Blood samples were collected by venipuncture, into sodium-heparin Vacutainer® tubes (BD Diagnostics). Samples were centrifuged for 10 min at 1500 g at 4 °C, within an hour of sampling, and the supernatant plasma was aliquoted into 1 ml polypropylene tubes and frozen at −80 °C.

Samples were sent to analyze by temperature regulated dry ice transport to the University of Gothenburg Mölndal, Sweden—Department of Psychiatry and Neurochemistry, Institute of Neuroscience and Physiology. Plasma samples of p-tau217 were measured using the NULISAseq (Alamar) CNS panel [30] according to the manufacturer's instructions. NULISA values are expressed in NULISA Protein Quantification (NPQ) units.

### Cognitive assessment

All individuals underwent extensive cognitive assessment. Briefly, six tests were used to derive four different cognitive domains. Verbal episodic memory was measured using the Rey Auditory Verbal Learning Test (RAVLT) and the free recall score after 30 min (RAVLT retention) tests [31]. The Rey-Osterrieth Complex Figure delayed recall (RO retention) was used to assess visuospatial episodic memory [31]. Executive function was assessed with both the Trail Making Test B (TMT-B) [31], and the Coding subtest from the Wechsler Adult Intelligence Scale – Fourth Edition (WAIS-IV) [32]. Finally, short term memory/attention was measured using Trail Making Test A (TMT-A) [31]. Participant neuropsychological testing scores were converted to Z-scores using the CN group as reference. Composite scores for each cognitive domain were calculated by averaging the Z-scores of the corresponding individual tests. Lower (i.e. more negative) Z-scores were representative of greater cognitive impairment. We used Mini-Mental State Examination (MMSE) [33] as a global cognitive measure. Mean time from neuropsychological testing to biomarker testing was 1.30 years (SD = 2.13).

### Statistical analyses

All analyses were carried out in R version 4.4.3 (https://www.r-project.org). Data visualization was conducted using *ggplot2* package, and regional brain maps were generated with *ggseg*. Participants with a non-AD diagnosis (MCI Aβ-) were excluded from the main analyses to ensure a homogeneous AD-focused sample. To determine whether analyzing the cognitively normal (CN) group as a whole across its full age range could introduce age-related bias, CN participants were stratified into younger (< 65 years) and older (≥ 65 years) subgroups. Differences in tau PET binding and plasma p-tau217 levels between these subgroups were assessed. As no significant differences were detected (Supplementary Table 1), CN participants were analyzed as a single group in subsequent analyses.

Group comparisons were performed using the Kruskal–Wallis test, with Wilcoxon rank-sum post hoc test for pairwise comparisons.

Following a similar procedure described in Cho et al. [25] regional tau involvement was determined first by computing Z-scores for each participant using the CN individuals as reference. Regions with Z-scores > 2 were classified as tau-positive. For each region, the proportion of participants showing involvement was calculated. Regions with higher frequency of tau positivity are interpreted as more commonly and/or earlier affected in the disease course. Sensitivity analyses were conducted using age-matched CN reference groups, applying the younger CN subgroup for EOAD and the older CN subgroup for LOAD. Tau PET and cognitive Z-scores were recalculated under this stratified approach and compared with those derived from the pooled CN reference group. Results were highly consistent across methods, with no significant differences observed. For a more detailed fine-grained differences in [^18^F]RO948 tau-PET signal voxel-wise group comparisons were performed with SPM12 using two sample t-tests. Only clusters surviving family-wise error (FWE) correction at *p* < 0.05 at the cluster level were considered statistically significant. First, we compared MCI Aβ-, MCI Aβ +, and dementia AD groups against CN participants. Then, subgroup comparisons were performed between Early- and Late-onset within both MCI and AD stages. Results were visualized using BrainNet Viewer with interpolated surface mapping [34].

Spearman correlations were performed with pairwise deletion. To assess whether the relationship between [^18^F]RO948 tau-PET and plasma p-tau217 differed by age at onset group (EOAD vs. LOAD)**,** we employed Generalized Additive Models (GAMs) including an interaction term (plasma × age group) [35]. GAMs were implemented using the *mgcv* package (method = "REML", family = Gamma (link = "log")). To maintain statistical stability and prevent the need to fit interaction models within small clinical subgroups, all GAMs were estimated on the full sample. To better model the age-related clinical span, younger CN individuals (age < 65) were included in the EOAD group curve, and older CN participants (age > 65) were included in the LOAD group curve. This resulted in age-stratified interaction curves comprising 22 younger and 25 older participants, respectively. To directly test whether the smooth functions differed between age at onset groups, Wald tests were conducted on the differences of the estimated smooth term coefficients [36]. Linear models were used to test whether tau PET or plasma p-tau217 differentially predicted CSF measures, cognitive outcomes or cortical thickness or volumes in EOAD and LOAD groups (e.g., region or plasma p-tau217 × age group). Age-group interaction analyses were exploratory in nature, and no multiple-comparison correction was applied. All analyses were adjusted for age, sex, and *APOE* status. Sensitivity analyses were performed comparing our primary models (adjusted for sex and APOE) with simplified models controlling only for age. Results were highly consistent across model specifications.

To control multiple comparisons in pairwise correlation analyses, false discovery rate (FDR) correction (Benjamini-Hochberg) was applied to the resulting *p*-values. Adjusted *p*-values < 0.05 were considered statistically significant.

## Results

### Participant characteristics

Table 1 presents characteristics of the individuals, including demographic, clinical, and biomarker data of all 57 participants which consisted of 18 cognitive healthy controls, 10 MCI Aβ-, 16 MCI Aβ + (9 MCI-EO and 7 MCI-LO), 13 AD patients (5 EOAD and 8 LOAD). Additional biomarker and cognitive data are also presented in Supplementary Table 2. Although not statistically significant, EOAD individuals showed poorer cognitive performance in episodic memory and visuospatial memory than LOAD but similar volumetric or thickness measures (Table 1 and Supplementary Table 2).

**Table 1:** Participant characteristics. Values are presented as Mean (SD) (continuous variables) or n (%) (categorical variables). Shown are demographic variables, APOE ε4 carriership, cerebrospinal fluid (CSF) and plasma phosphorylated tau (p-tau217), tau-PET regional Standardized Uptake Value Ratio (SUVR), cortical thickness measures, and cognitive Z-scores across cognitively normal (CN), mild cognitive impairment amyloid-negative (MCI Aβ–), mild cognitive impairment amyloid-positive (MCI Aβ +), and Alzheimer’s disease (AD) groups, for both early-onset (EOAD) and late-onset (LOAD) cases.

Variable	CN *N* = 18	MCI Aß- *N* = 10	MCI Aß + *N* = 16	AD *N* = 13	MCI Aß + EO *N* = 9	EOAD *N* = 5	MCI Aß + LO *N* = 7	LOAD *N* = 8
Age (years)	65 (9)	78 (3)	68 (8)	71 (10)	61 (5)	61 (3)	75 (3)^c^	77 (6)^d^
Sex (%)								
Female	8 (44%)	3 (30%)	9 (56%)	6 (46%)	5 (56%)	2 (40%)	2 (29%)	5 (63%)
Male	10 (56%)	7 (70%)	7 (44%)	7 (54%)	4 (44%)	3 (60%)	5 (71%)	3 (38%)
Education (years)	12.59 (3.00)	15.00 (2.16)	15.21 (1.47)ᵃ	13.63 (2.45)	14.17 (1.61)	12.33 (0.58)	16.00 (0.82)	14.40 (2.88)
MMSE	28.61 (1.38)	27.33 (1.58)	26.00 (4.08)	24.46 (3.07)ᵃ	26.00 (3.46)	23.60 (2.07)	26.00 (4.90)	25.00 (3.59)
APOE E4 carriership (%)								
E4	4 (22%)	2 (20%)	13 (81%)ᵃ	11 (85%)ᵃ	7 (78%)	5 (100%)	6 (86%)	6 (75%)
CSF p-tau181		48 (17)	79 (45)	112 (68)	70 (47)	119 (67)	90 (44)	107 (73)
CSF Aβ42/40		0.99 (0.17)	0.54 (0.12)	0.47 (0.06)	0.57 (0.12)	0.45 (0.06)	0.51 (0.12)	0.49 (0.06)
MTA								
0–1		5 (50%)	10 (63%)	8 (62%)	7 (78%)	3 (60%)	3 (43%)	5 (63%)
2		1 (10%)	6 (38%)	4 (31%)	2 (22%)	2 (40%)	4 (57%)	2 (25%)
3–4		4 (40%)	0 (0%)	1 (7.7%)	0 (0%)	0 (0%)	0 (0%)	1 (13%)
Plasma p-tau217	11.03 (0.39)	11.60 (0.32)	12.10 (0.45)ᵃ	12.66 (0.38)ᵃᵇ	11.94 (0.52)	12.55 (0.34)	12.31 (0.22)	12.72 (0.41)
Entorhinal Cortex SUVR	1.23 (0.14)	1.31 (0.19)	1.91 (0.42)ᵃ	2.21 (0.41)ᵃ	1.91 (0.54)	2.07 (0.52)	1.92 (0.24)	2.29 (0.34)
Inferior Temporal Cortex SUVR	1.35 (0.15)	1.32 (0.12)	1.83 (0.65)ᵃ	2.29 (0.71)ᵃ	1.90 (0.80)	2.41 (0.96)	1.75 (0.44)	2.22 (0.58)
Parietal Cortex SUVR	1.19 (0.12)	1.16 (0.14)	1.41 (0.48)	1.84 (0.66)ᵃᵇ	1.58 (0.59)	2.39 (0.72)	1.19 (0.09)	1.50 (0.34)
Attention Z-score	0.00 (1.00)	−3.80 (2.23)	−3.52 (2.16)ᵃ	−3.33 (2.03)	−4.18 (2.55)	−2.05 (2.21)	−2.43 (0.66)	−3.96 (1.91)
Episodic Memory z-score	0.00 (0.95)	−1.22 (0.70)	−1.26 (1.64)ᵃ	−1.58 (0.78)	−1.30 (1.48)	−2.26 (0.19)	−1.19 (2.06)	−0.91 (0.42)
Visuospatial Memory Z-score	0.00 (1.00)	−1.32 (1.21)	−0.92 (1.37)	−1.67 (0.98)	−1.23 (1.19)	−2.11 (1.15)	−0.49 (1.61)	−1.34 (0.84)
Executive Function z-score	0.03 (0.85)	−1.72 (1.24)	−1.46 (1.73)ᵃ	−1.90 (1.27)	−1.57 (2.06)	−1.70 (1.83)	−1.28 (1.23)	−2.07 (0.80)
Entorhinal Cortex Thickness	3.32 (0.26)	2.81 (0.31)	2.96 (0.29)ᵃ	2.73 (0.34)	3.00 (0.31)	2.87 (0.33)	2.91 (0.28)	2.64 (0.34)
Inferior Temporal Cortex Thickness	2.68 (0.12)	2.60 (0.22)	2.57 (0.17)	2.54 (0.15)	2.57 (0.19)	2.57 (0.23)	2.57 (0.15)	2.53 (0.09)
Parietal Cortex Thickness	2.25 (0.09)	2.15 (0.09)ᵃ	2.25 (0.11)ᵇ	2.11 (0.14)	2.26 (0.13)	2.02 (0.18)	2.25 (0.08)	2.16 (0.08)

Group comparisons displayed: CN vs MCI Aβ + vs AD, MCI Aβ + -EO vs MCI Aβ + -LO, EOAD vs LOAD

a = statistically different from CN, b = statistically different from MCI Aβ +

c = statistically different from MCI Aβ + -EO, d = statistically different from EOAD. (*p* < 0.05)

Pathological thresholds for CSF-pTau181: 56.6 pg/ml and for CSF Aβ42/40: 0.68 pg/ml. MMSE = Mini-Mental State Examination

### Regional differences in [^18^F]RO948 binding across sporadic Alzheimer’s disease spectrum

#### Voxel-wise and regional analyses

**Fig. 1:**
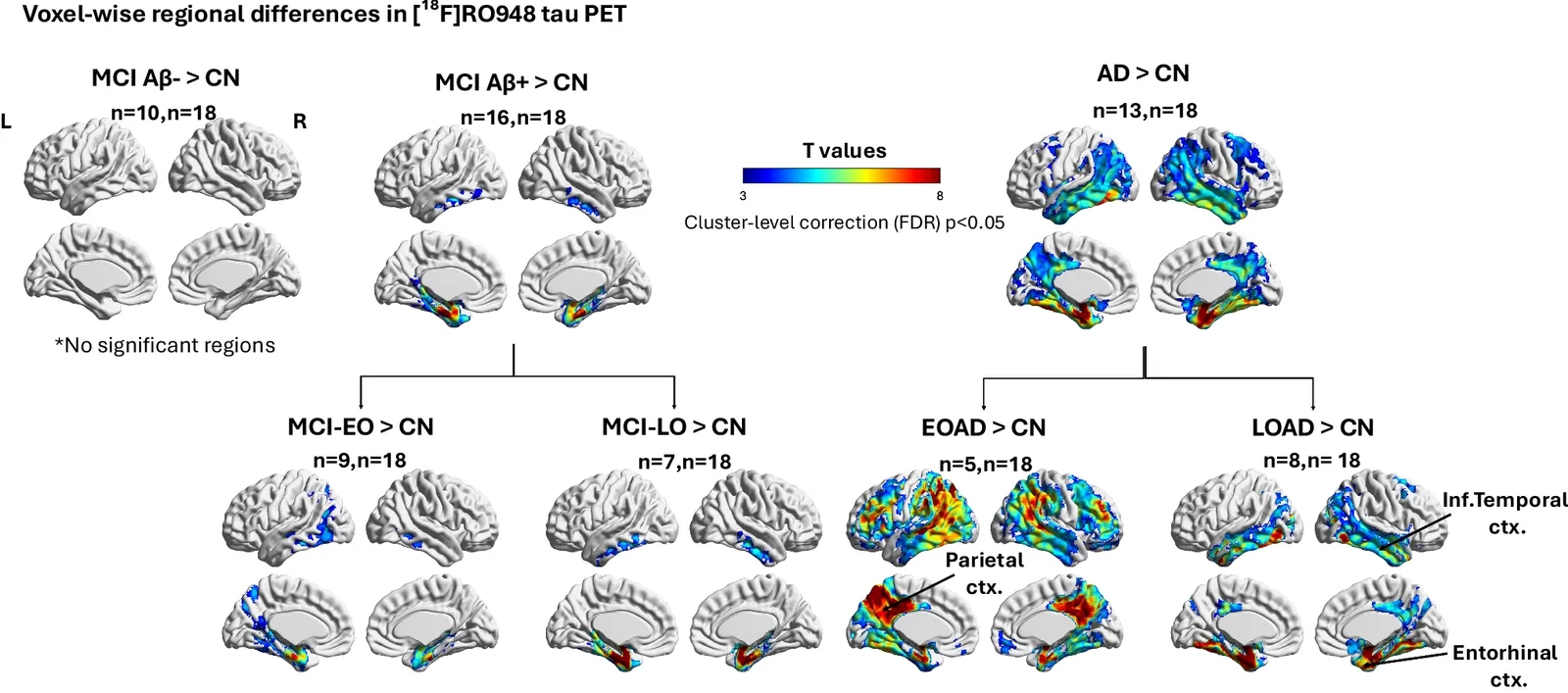
Voxel-wise differences in [^18^F]RO948 tau PET Standardized Uptake Value Ratio (SUVR) across Alzheimer’s disease diagnostic groups. Statistical parametric maps showing voxel-wise *t*-tests of [^18^F]RO948 tau-PET Standardized Uptake Value Ratio (SUVR) between each clinical group and the Cognitively Normal (CN) reference group. The voxel-wise t-tests were corrected at a cluster level with the FDR method at *p* < 0.05

**Fig. 2:**
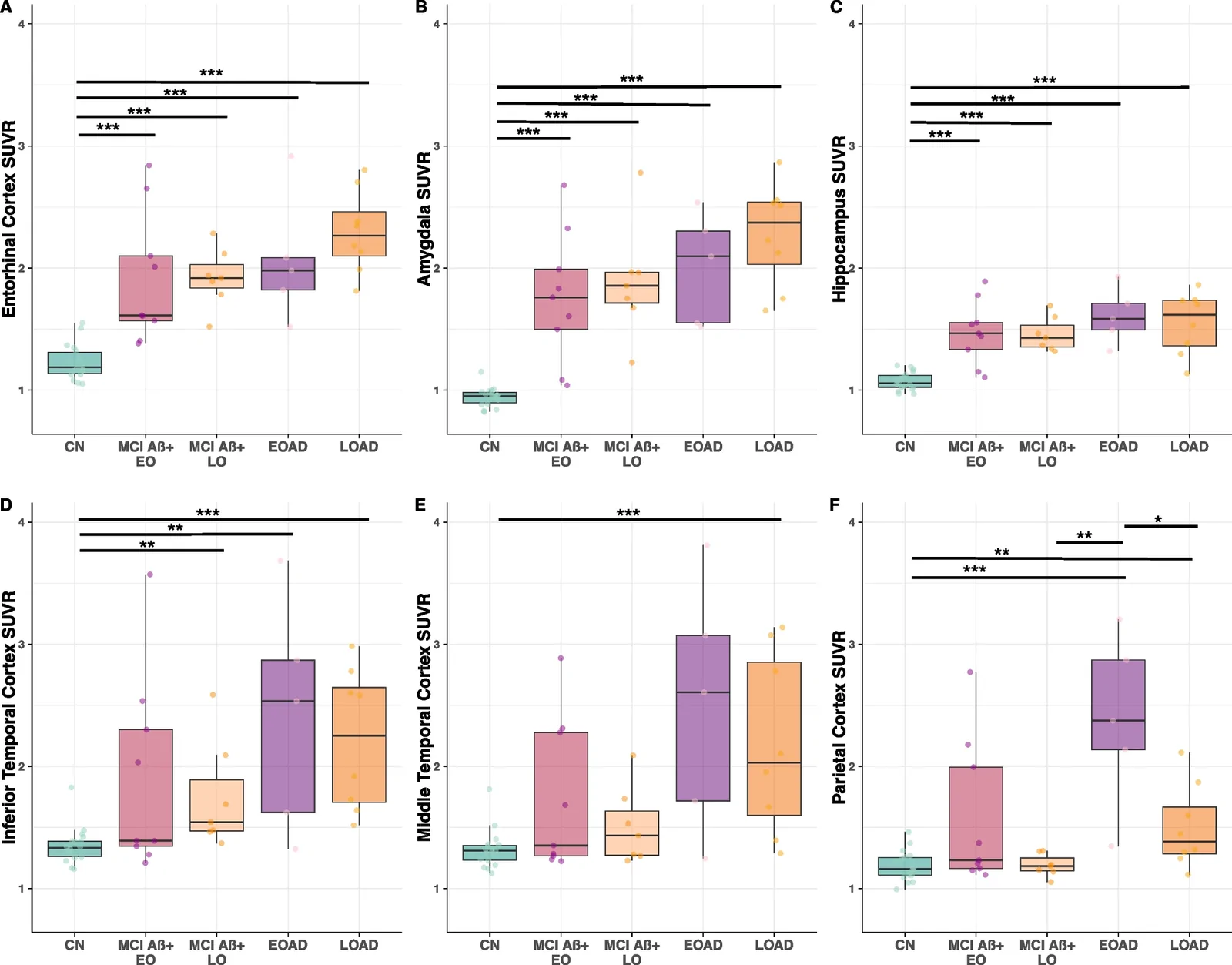
Regional-specific differences in [¹⁸F]RO948Comment tau PET Standardized Uptake Value across Alzheimer’s disease diagnostic groups. Boxplots showing Standardized Uptake Value Ratio (SUVR) values in the selected regions across cognitively normal (CN), Mild Cognitive Impairment amyloid-positive (MCI Aβ +) and Alzheimer’s Disease (AD) groups in both early-onset (EO) and late-onset (LO) Alzheimer’s disease. Asterisks indicate significance levels from pairwise comparisons (*p < 0.05; **p < 0.01; ***p < 0.001)

Figure 1 show the voxel-wise [^18^F]RO948 tau-PET binding across AD diagnostic groups. MCI Aβ + individuals exhibited significantly higher tau-PET SUVRs in subcortical and medial-temporal areas, specifically region-based analyses showed increased SUVRs in the entorhinal cortex, amygdala, and hippocampus (all *p* < 0.001), and extended also to inferior temporal region (*p* < 0.01) relative to cognitively normal (CN) participants (Fig. 1 and Supplementary Fig. 2). No significant differences in these regions were observed between MCI-EO and MCI-LO (Figs. 1 and 2).

AD individuals showed higher tau-PET binding in temporo-parietal regions than CN and MCI Aβ + (Fig. 1 and Supplementary Fig. 2), but group-specific differences emerged: the EOAD group showed greater neocortical extent involving temporo-parietal and frontal cortex compared to CN, whereas LOAD tau PET uptake remined in the temporal areas (Fig. 1). Specifically, parietal binding was significantly higher in EOAD than LOAD (*p* = 0.046) (Fig. 2). No differences were found in tau-PET SUVR between MCI Aβ- and CN groups. Region-specific tau-PET SUVRs are provided in Table 1 and Supplementary Table 2.

#### Individual tau-PET profiles

**Fig. 3:**
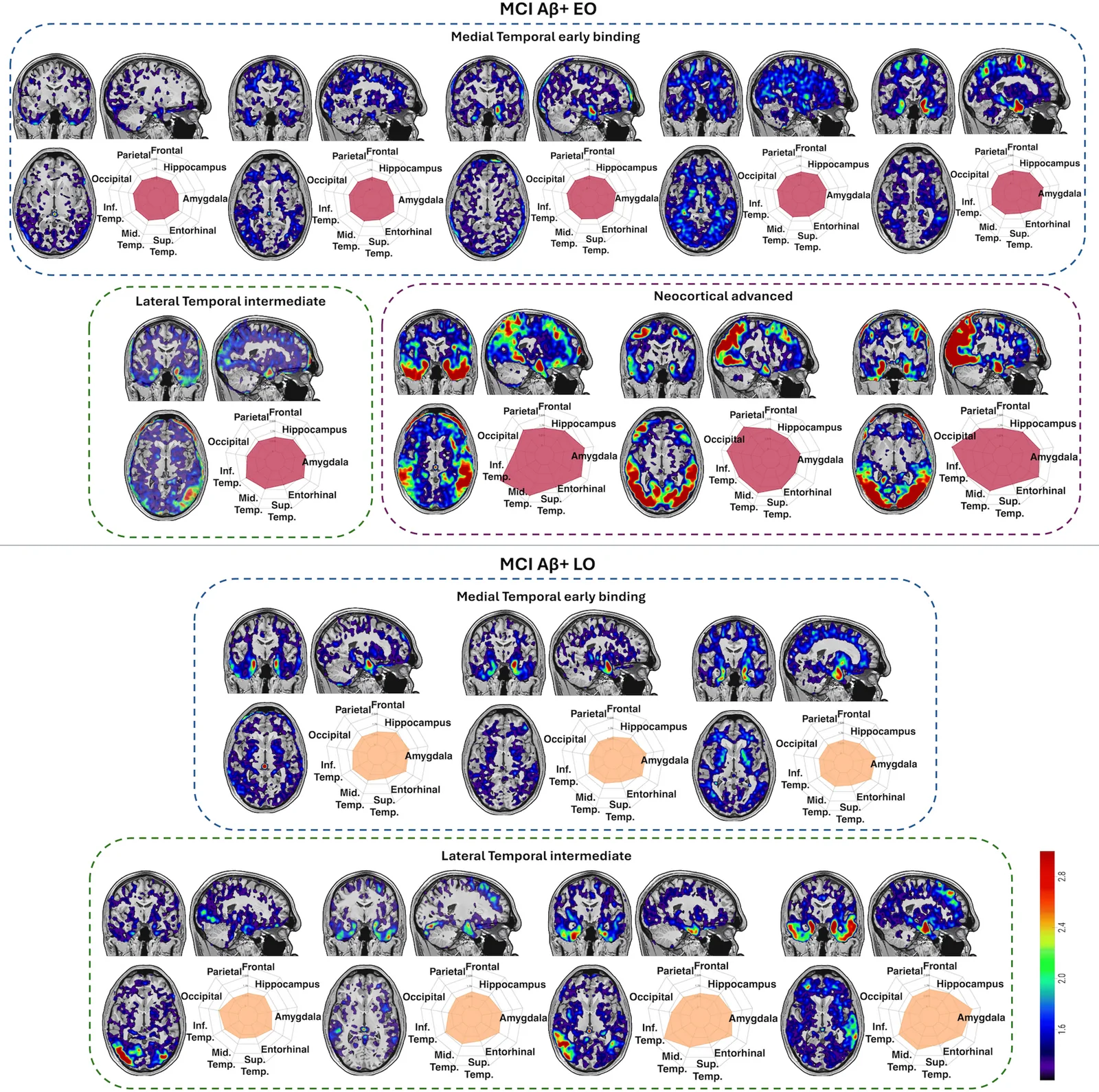
[^18^F]RO948 tau binding distribution in MCI groups by age at onset. [^18^F]RO948 tau PET scans (overlayed on corresponding MRI for anatomical reference) for Mild Cognitive Impairment amyloid-positive (MCI Aβ +) individuals are shown and subdivided according to early-onset (MCI-EO) and late-onset (MCI-LO) Alzheimer’s disease subgroups. For each subject, coronal, sagittal and axial views and radar plots display Standard Uptake Value Ratio (SUVR) values across key brain regions (Frontal, Parietal, Occipital, Inferior Temporal, Middle Temporal, Superior Temporal, Entorhinal, Amygdala, Hippocampus)

Figure 3 shows individual tau-PET scans in the 9 MCI-EO and 7 MCI-LO patients, illustrating marked variability in binding across the MCI Aβ + group. Individuals were categorized according to the regional extent of [^18^F]RO948 binding, reflecting progressive stages of tau accumulation from medial temporal to neocortical regions. Among MCI-EO individuals, five showed early binding in the medial-temporal regions, one displayed lateral temporal intermediate binding, and three exhibited advanced neocortical involvement. In contrast, MCI-LO patients showed three cases with medial temporal early binding and four with lateral temporal intermediate tau-PET binding. Overall, MCI-EO cases exhibited a greater spatial extent and burden of tau than MCI-LO (Fig. 3); this was also evident in EOAD vs LOAD individuals (Supplementary Fig. 3).

#### Regional prevalence of abnormal tau-PET binding

**Fig. 4:**
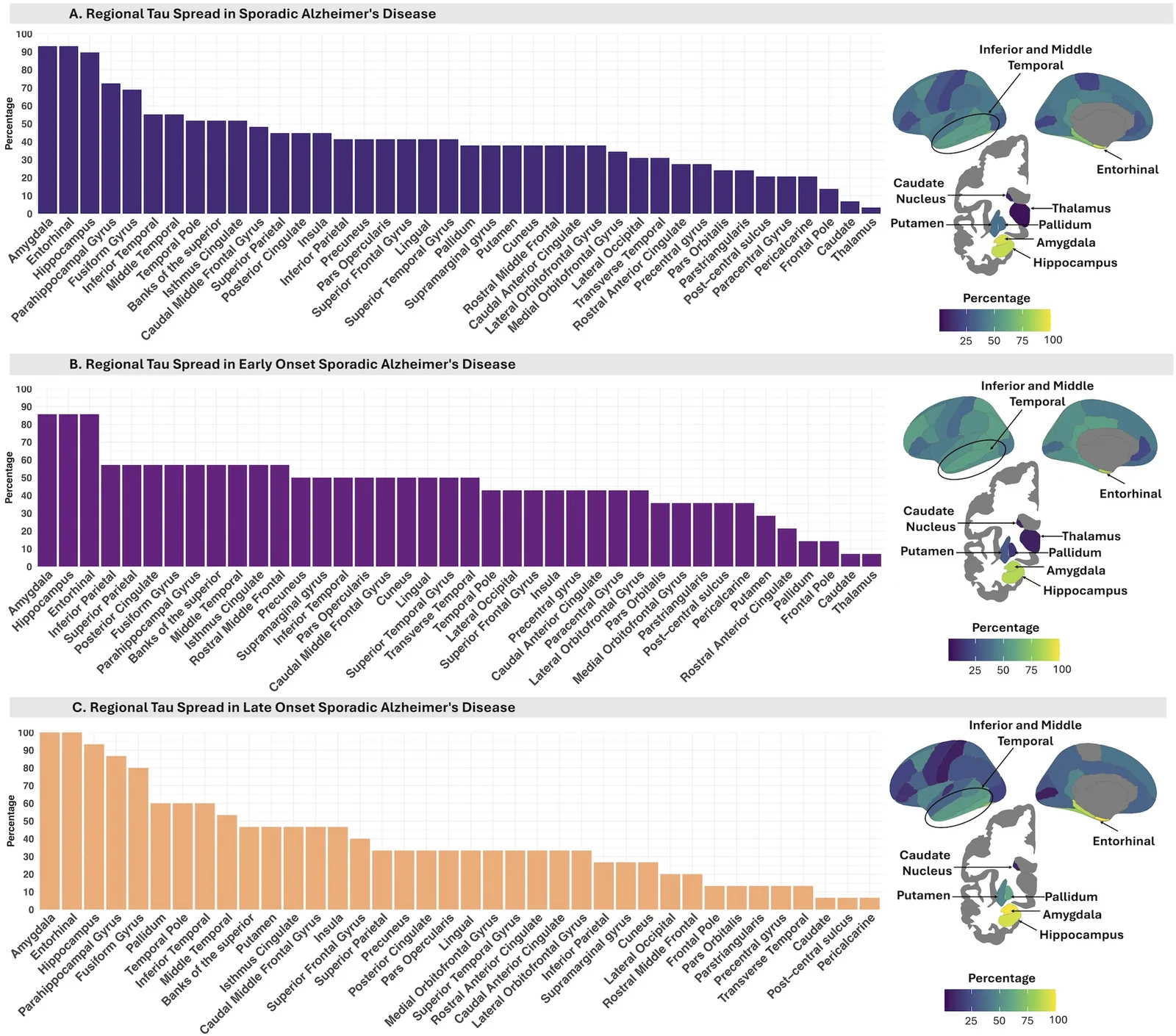
Regional Tau Extent in Sporadic Alzheimer’s Disease. The figure illustrates the percentage of individuals with abnormal [^18^F]RO948 tau PET SUVRs across different brain regions, based on Z-scores > 2 relative to cognitively normal (CN) controls. Top bar plots show the overall percentage of regional tau involvement among participants with Mild Cognitive Impairment (MCI) and Alzheimer’s Disease (AD). The middle and bottom panels further divide the cohort into early-onset and late-onset subgroups. On the right, corresponding brain maps display the spatial distribution of regional tau involvement. Brighter colors indicate a higher proportion of individuals with tau-PET positivity in each region

When we defined regional abnormal tau signal as Z-scores > 2 relatively to CN individuals the MCI Aβ + and AD individuals showed highest percentage (≈90%) of abnormal [^18^F]RO948 tau-PET SUVR predominantly in the medial-temporal and subcortical areas (i.e. the entorhinal cortex, the amygdala and the hippocampus) (Fig. 4A). Overall, EOAD demonstrated -more frequently- abnormally high tau-PET binding in several brain areas (≥ 50% in several areas) compared with LOAD with most of the impaired areas confined to temporal cortex (Fig. 4B-C).

### Plasma p-tau217 and its association with tau PET by age at onset subtypes

**Fig. 5:**
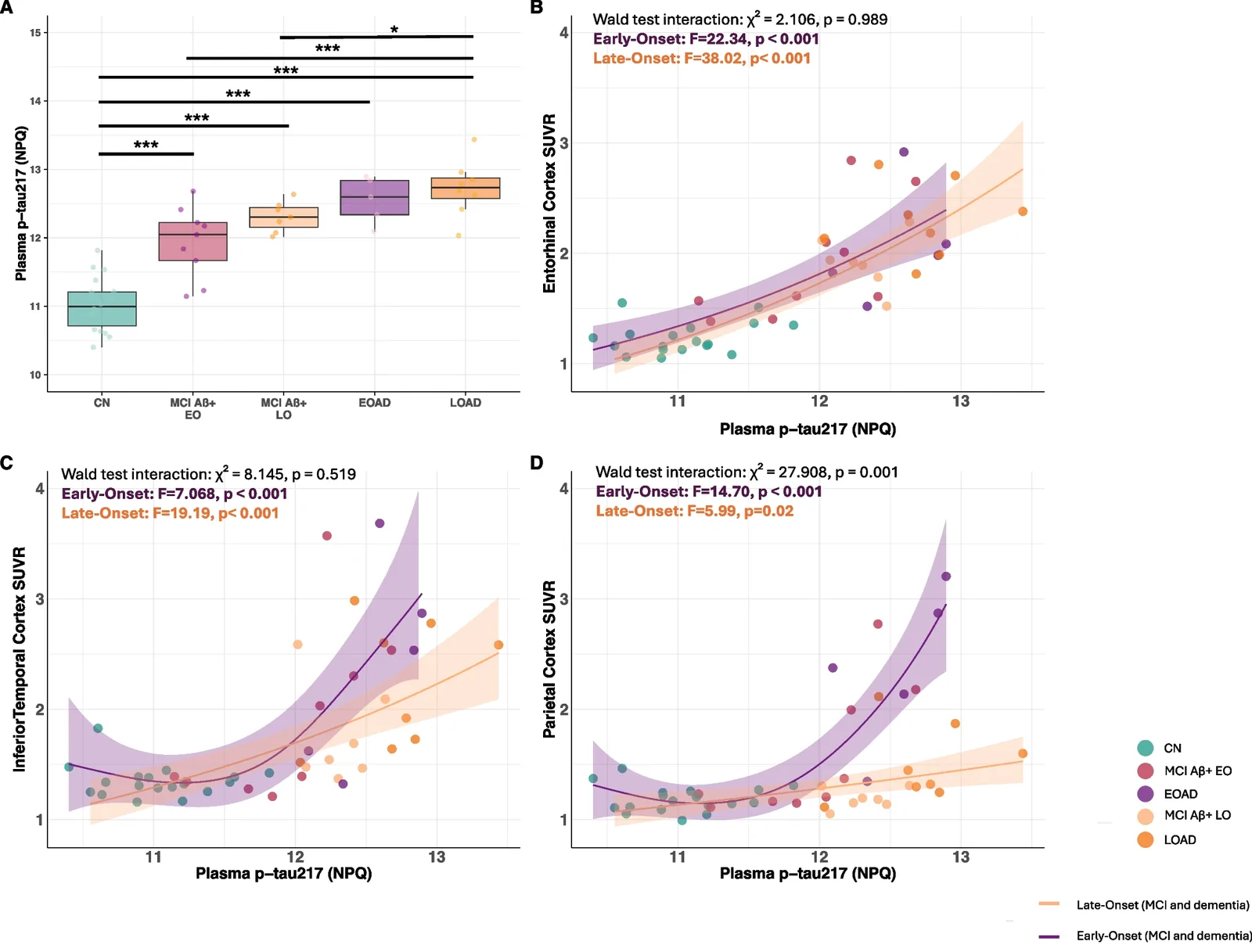
Plasma p-tau217 differences across Alzheimer’s disease diagnostic groups and its relationship with tau PET. Boxplots show the distribution of plasma p-tau217 across Alzheimer’s Disease diagnostic groups: cognitively normal (CN), early-onset and late-onset with Mild Cognitive Impairment (MCI Aβ +) or Alzheimer’s Disease (AD) (Panel **A**). Individual data points are shown with overlaid boxplots representing the median and interquartile range (IQR); whiskers extend to 1.5 × IQR. Horizontal lines indicate significant pairwise comparisons between groups based on post hoc tests, adjusted for multiple comparisons. Significance levels are denoted as follows: (*p < 0.05; **p < 0.01; ***p < 0.001). (Panels **B**-**D**) Regressions between plasma p-tau217 and regional tau PET binding in the entorhinal (**B**), inferior temporal cortex (**C**), and parietal cortex (**D**), modeled using generalized additive models (GAMs) with a gamma distribution and log-link function. Separate smooth terms are fitted for early-onset MCI and AD (purple line) and late-onset MCI and AD (orange line). Shaded areas represent 95% confidence intervals. Wald tests of the difference between the smoothed curves for EOAD and LOAD are also shown (chi-squared and *p*-value). NULISA biomarkers are expressed in NULISA Protein Quantification (NPQ) units. All GAMs analyses were controlled for Sex and APOE status as covariates. p-tau217 = phosphorylated tau 217, SUVR = Standardized Uptake Value Ratio

Plasma p-tau217 levels were higher in both EOAD and LOAD (MCI Aβ + and AD) compared with cognitively normal controls (all *p* < 0.05). Plasma p-tau217 differentiated MCI from AD in LOAD, but not in EOAD (Fig. 5A and Table 1).

Stronger positive correlations were found between plasma p-tau217 levels and tau PET in subcortical and medial-temporal regions (i.e., the amygdala, the entorhinal cortex, and the hippocampus), followed by the temporal and parietal cortices (*all p* < 0.001) (Supplementary Fig. 4). GAM curves indicated similar association patterns in the subcortical, medial-temporal and inferior temporal regions for both EOAD and LOAD groups, while parietal regions showed marked differences between groups, confirmed by Wald tests (Fig. 5B–D). Results for the GAM interaction models, including both age-only adjusted and totally adjusted versions across all brain regions, are reported in Supplementary Table 3.

Tau-PET showed a significant positive correlation with CSF p-tau181 (entorhinal cortex: *partial rho* = 0.52; *p* = 0.019), whereas plasma p-tau217 did not show meaningful associations with CSF. No differences between EOAD and LOAD were found. (Supplementary Fig. 4).

### Cross-sectional associations between tau-PET and plasma p-tau217 with cognition by age at onset subtypes

**Fig. 6:**
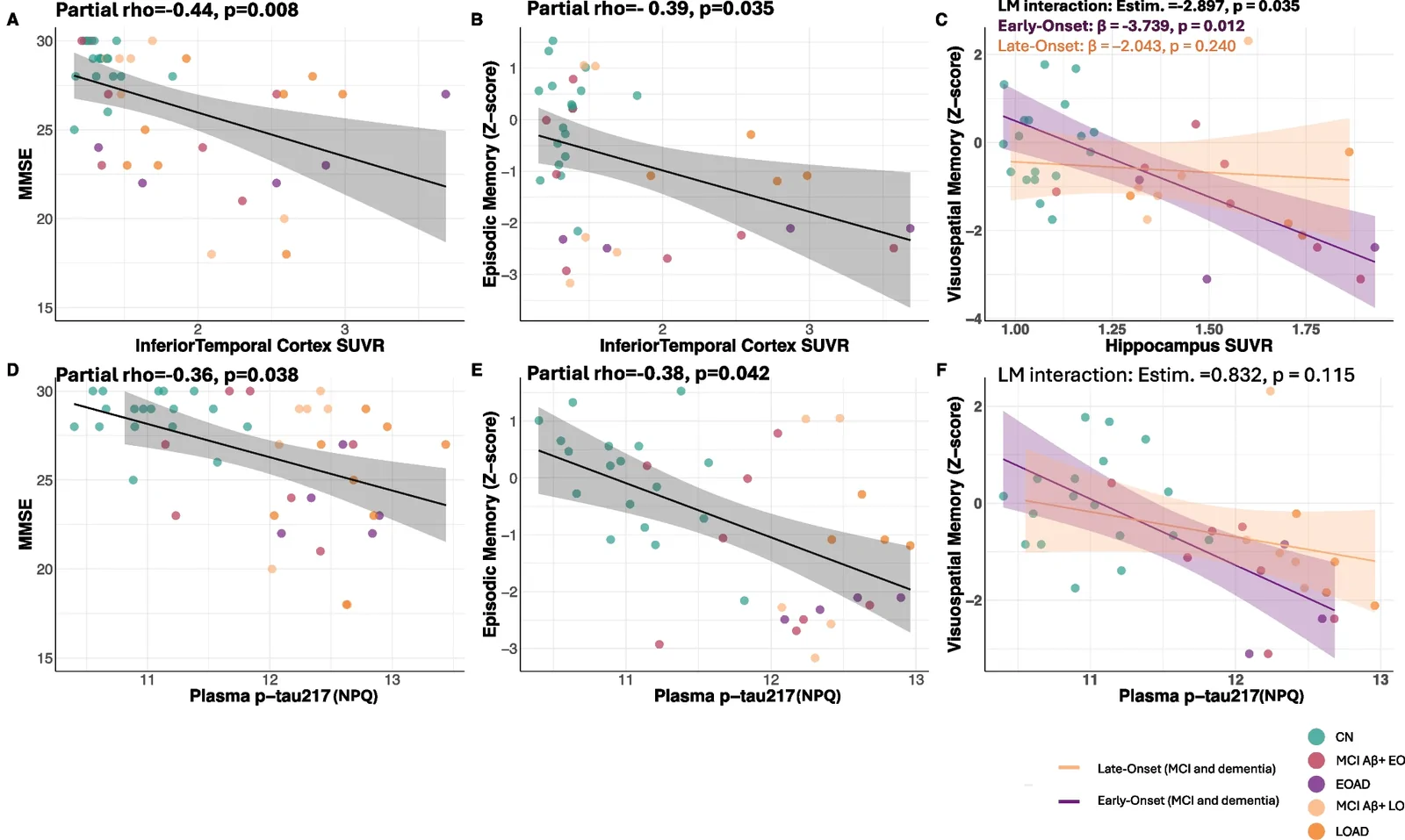
Regional correlations between [^18^F]RO948 tau PET binding, plasma biomarkers and cognition. (Panels **A**-**C**) Correlations between Inferior Temporal [^18^F]RO948 Standard Uptake Value Rario (SUVR) and (**A**) Mini-mental State Examination (MMSE), (**B**) Episodic Memory, (**C**) and Hippocampus [^18^F]RO948 SUVRs and Visuospatial memory. (Panels **D**-**F**) Correlations between plasma phosphorylated tau (p-tau217) and (**D**) MMSE, (**E**) Episodic Memory, (**F**) Visuospatial memory. MMSE (*n* = 45), Episodic Memory Z-score(*n* = 39) and Visuospatial Memory Z-score (*n* = 36). Scatter plots show raw data points and unadjusted regression lines. Shaded areas represent 95% confidence intervals. Partial correlation coefficients (rho) and *p*-values are derived from models controlling for Sex and APOE status. Panels (**C**, **F**) fitted lines are presented by EOAD/LOAD and LM interaction estimates and *p*-values are derived from models controlling for Sex and APOE status. NULISA plasma p-tau217 levels are expressed in NULISA Protein Quantification (NPQ) units

Tau PET binding was negatively associated with all cognitive domains except attention, particularly in the inferior temporal and parietal cortices (*all p* < 0.05; Table 2, Fig. 6A–B). In EOAD, amygdala (β = –1.78, *p* = 0.013) and hippocampal (β = –3.74, p = 0.012) tau binding was associated with visuospatial memory, whereas these associations were not observed in LOAD (Table 2, Fig. 6C). No other differences were found (Supplementary Fig. 5). Visual inspection of individual tau-PET SUVR maps showed marked heterogeneity in tau burden within both EOAD and LOAD, reflected in divergent cognitive profiles. EOAD cases displayed higher temporoparietal tau uptake and poorer visuospatial performance (Supplementary Fig. 6).

**Table 2:** Partial correlations between regional [^18^F]RO948 Standard Uptake Value Ratio (SUVRs) and plasma p-tau217 levels with cognitive outcomes. Partial correlation coefficients (rho) and *p*-values are derived from models controlling for Sex and APOE status.

	Inferior Temporal		Parietal		Plasma p-tau217		Amygdala*Age group		Hippocampus*Age group		Plasma p-tau217*Age group	
Outcome	Partial rho	*P*-value	Partial rho	*P*-value	Partial rho	*P*-value	Estim.	*P*-value	Estim.	*P*-value	Estim.	*P*-value
MMSE	−0.44	**0.008**	−0.41	**0.017**	−0.36	**0.038**	−0.200	0.898	−1.477	0.667	0.285	0.814
Episodic Memory Z-score	−0.39	**0.035**	−0.40	**0.030**	−0.38	**0.042**	0.679	0.288	1.718	0.240	0.775	0.131
Executive Function Z-score	−0.41	**0.023**	−0.42	**0.019**	−0.27	0.157	−0.277	0.713	0.042	0.980	−0.053	0.928
Attention Z-score	−0.22	0.346	−0.07	0.840	−0.36	0.087	−0.774	0.562	−1.785	0.527	0.217	0.825
Visuospatial Memory Z-score	−0.55	**0.003**	−0.58	**0.001**	−0.41	**0.028**	1.414	**0.024**	2.897	**0.035**	−0.832	0.115

Interaction effects between age group (EOAD/LOAD) and regional [^18^F]RO948 Standard Uptake Value Ratio (SUVRs) or plasma p-tau217 are shown as regression estimates (Estim.) with corresponding *p*-values from models controlling for Sex and APOE status. Significant associations (*p* < 0.05) are highlighted in bold. MMSE(*n* = 45), Episodic Memory Z -score(*n* = 39) Visuospatial Memory Z-score (*n* = 36), Executive Function Z-score (*n* = 40), and Attention Z-score(*n* = 32)

Plasma p-tau217 showed negative correlations with MMSE, verbal and visuospatial episodic memory (all *p* < 0.05), but not with executive function or attention (Table 2, Fig. 6D–E). No significant differences in plasma–cognition associations were observed between EOAD and LOAD (Table 2; Fig. 6 F, Supplementary Fig. 5).

### Cross-sectional associations between tau-PET and plasma p-tau217 with brain atrophy by age at onset subtypes

**Fig. 7:**
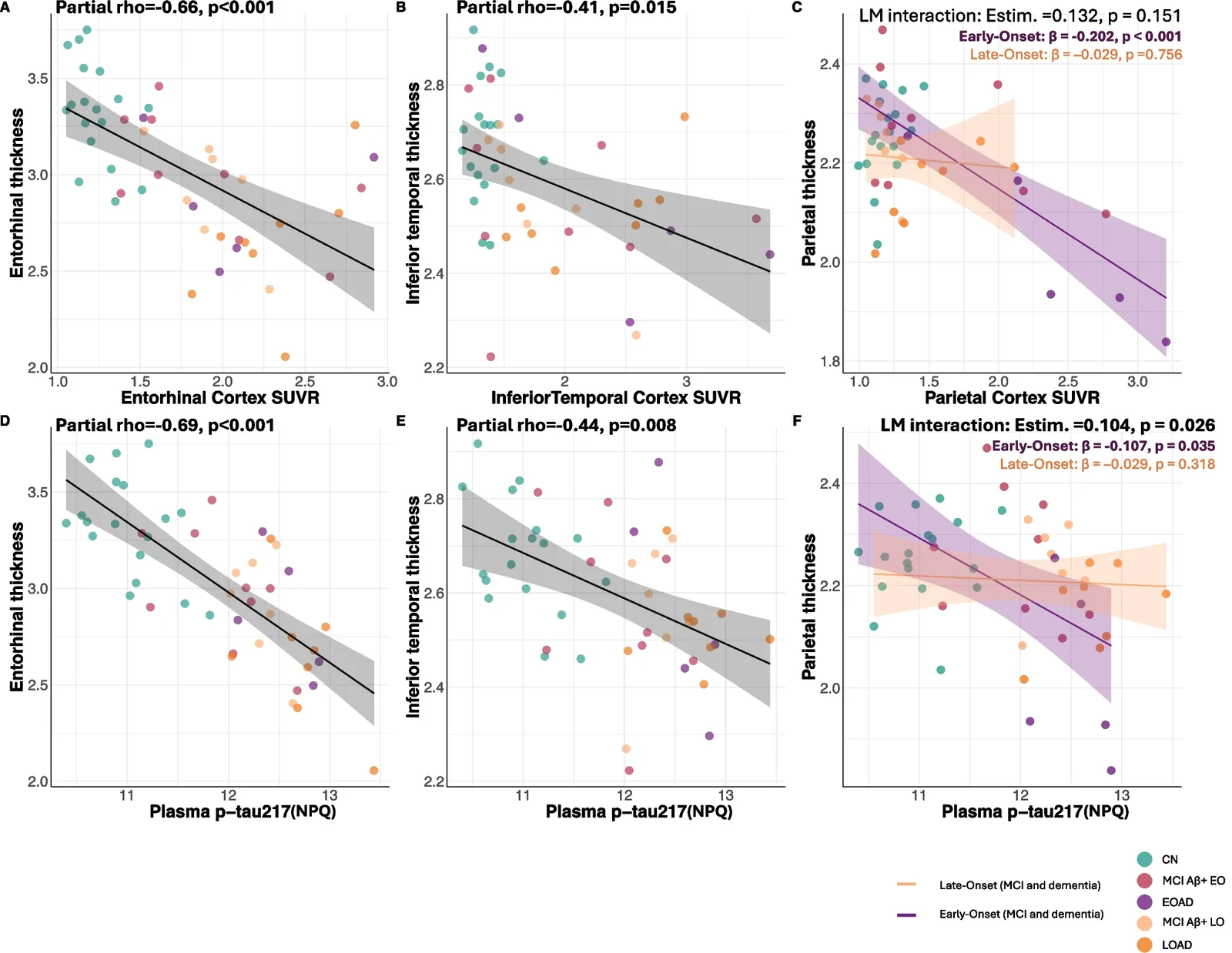
Regional correlations between [^18^F]RO948 tau PET binding, plasma biomarkers and cortical thickness. Panels (**A-C**) Correlations between: (**A**) Entorhinal [^18^F]RO948 SUVR and Entorhinal thickness, (**B**) Inferior Temporal [^18^F]RO948 SUVR and Inferior Temporal thickness, (**C**) Parietal [^18^F]RO948 SUVR and Parietal thickness, (Panels **D-F**) Correlations between plasma phosphorylated tau (p-tau217) and: (**D**) Entorhinal thickness, (**E**) Inferior Temporal thickness, (**F**) Parietal thickness. Scatter plots show raw data points and unadjusted regression lines. Shaded areas represent 95% confidence intervals. Partial correlation coefficients (rho) and *p*-values are derived from models controlling for Sex and APOE status. Panel (**C**, **F**) fitted lines are presented by EOAD/LOAD and LM interaction estimates and *p*-values are derived from models controlling for Sex and APOE status. NULISA plasma p-tau217 levels are expressed in NULISA Protein Quantification (NPQ) units. Group abbreviations: C*N* = cognitively normal; MCI-EO/MCI-LO = early-onset and late-onset disease-Mild Cognitive Impairment; EOAD/LOAD = early-onset and late-onset Alzheimer’s disease

Similar negative correlations were observed between cortical thickness or volume measures and tau-PET or plasma p-tau217. Both tau-PET measures and plasma p-tau217 values showed significant negative correlations with medial-temporal and lateral temporal MRI measures (*all p* < 0.05). Lower parietal thickness was negatively associated with higher parietal tau-PET SUVRs (*β* = −0.202, *p* = 0.001) and higher plasmaZ p-tau217 (*β* = −0.107, *p* = 0.035) in the EOAD group specifically (Fig. 7). No other significant differences between EOAD vs LOAD were found in the tau-PET/plasma-thickness relationships (Supplementary Fig. 7).

## Discussion

In this study, we characterized the regional extent of tau pathology using the second-generation tau-PET tracer [^18^F]RO948 across the Alzheimer’s disease spectrum, with a particular focus on age at onset (EO vs LO). Although differences in tau burden have been linked to disease stage and age at onset, it remains unclear how such variation is reflected in tau-PET and plasma p-tau217. To our knowledge, this is the first study to specifically assess tau PET–plasma relationships in the context of age at onset. We found that EO individuals exhibited greater neocortical tau involvement even at prodromal stages along with a more widespread pattern of tau distribution in AD stages compared to LO. These differences were not consistently mirrored by plasma p-tau217 levels, as similar plasma values were associated with substantial variability in neocortical tau-PET binding across individuals. Notably, in LOAD, high plasma p-tau217 levels did not necessarily correspond to high tau-PET uptake in neocortical regions. These findings further support the sensitivity of [^18^F]RO948 for detecting early and regionally heterogeneous tau pathology.

We observed early tau accumulation in MCI Aβ + individuals compared to cognitively normal participants, indicating that tau pathology associated with neurofibrillary tangles can be detected at earlier clinical stages than previously visualized by imaging. Specifically, subcortical structures such as the amygdala and hippocampus were involved at the prodromal stage, in line with postmortem evidence identifying these areas as initial sites of neurofibrillary tangle formation [3, 4, 37, 38]. These results support [^18^F]RO948 as a sensitive tracer capable of detecting initial stages of tau aggregation in the brain, including regions such as the hippocampus, where first-generation tracers like [^18^F]AV-1451 (Flourtaucipir) have shown limited signal-to-noise ratio [16, 22, 39]. The improved sensitivity of [1⁸F]RO948 and other second-generation tracers (e.g., [^18^F]MK6240) enhances discrimination at the prodromal stage of AD, where tau accumulation is subtle. In contrast, [^18^F]Flortaucipir shows substantially lower discriminative accuracy at this stage due to less pronounced tau binding in MCI than in AD dementia [40]. Beyond the canonical Braak trajectory [3, 41, 42], we identified a distinct topographical influence of age at onset: while both MCI-EO and MCI-LO showed elevated tau in medial temporal regions, EOAD cases exhibited greater neocortical involvement even at the MCI stage. These findings align with prior reports of regional heterogeneity by age at onset [11, 27, 43, 44], and underscore the value of second-generation tau-PET tracers like [^18^F]RO948 for mapping spatially variable pathology in vivo.

Recent efforts have explored whether plasma biomarkers, particularly p-tau217, can serve as surrogates for tau-PET in clinical settings for early detection [45], patient stratification [46] and disease monitoring. We found that plasma p-tau217 levels increased across diagnostic stages, differentiating CN, MCI Aβ +, and AD. Notably, this stage-dependent increase was more pronounced in LOAD, whereas EOAD showed less separation between MCI and AD, suggesting age-at-onset–related differences in the dynamic range of plasma p-tau217 across disease stages [48]. Emerging evidence indicates that most of the variance in plasma p-tau217 levels is explained by amyloid-β pathology, whereas the remaining variance may reflect individual differences in tau accumulation [47, 48]. Extending these findings, we found that plasma p-tau217 did not differ significantly between MCI-EO and MCI-LO, nor between EOAD and LOAD groups. This contrasts with our tau-PET findings, which showed qualitatively higher SUVRs in MCI-EO. This apparent dissociation aligns with the regional correlation analyses: our results showed that plasma p-tau217 correlated most strongly with tau-PET in early-affected regions (the entorhinal cortex, hippocampus and amygdala). In later-affected neocortical regions—particularly the parietal cortices—the relationship becomes less significant and dependent on age at onset. In regions such as the inferior temporal cortex, higher plasma p-tau217 levels were generally associated with higher tau-PET uptake in both EOAD and LOAD; however, similar plasma values corresponded to different levels of tau burden across groups, indicating a shift in the plasma–PET relationship. This divergence was even more pronounced in parietal regions, where plasma p-tau217 remained associated with tau-PET in EOAD, but not in LOAD, where elevated plasma levels were observed even in individuals with relatively low tau-PET uptake [49]. These results suggest that EOAD and LOAD differential tau accumulation and load is not fully captured by plasma biomarkers. This finding has important implications for clinical treatment and participant stratification, as relying solely on plasma biomarkers could lead to misclassification of regional tau burden. Similar plasma p-tau 217 levels may reflect markedly different neocortical tau extent depending on age at onset. Our findings suggest that plasma p-tau217 capture early, amyloid-driven phosphorylation dynamics, but it does not fully match the spatial extent of tau pathology measured by tau-PET —highlighting the continued value of tau-PET for accurate disease stratification and for assessing therapeutic efficacy in individuals with distinct regional tau burdens [49].

In this study, we investigated cognitive symptoms across distinct domains and found that both tau-PET and plasma p-tau217 correlated with cognitive performance and cortical thickness, yet tau-PET showed stronger, domain-specific associations. While previous longitudinal studies demonstrated that tau-PET more closely tracks cognitive decline than plasma biomarkers [50], supporting its role as a marker of downstream neurodegeneration [6], our findings extend this work by demonstrating age-at-onset–specific patterns. In EOAD, tau accumulation in the middle temporal and parietal cortices corresponded to visuospatial impairment—an EOAD-typical cognitive feature [51, 52]. In contrast, plasma p-tau217 related broadly to global cognition (MMSE) and episodic memory (verbal, and visuospatial), with no differences between EOAD and LOAD, reflecting its link to early-phase pathology [50, 53, 54]. Together, these findings highlight the complementary but distinct roles of plasma and imaging biomarkers, with tau-PET providing greater spatial specificity for mapping cognitive phenotypes.

A major strength of this work is that it is the first to directly assess plasma–tau-PET–cognition relationships using [^18^F]RO948 in both early-onset and late-onset Alzheimer's disease across the AD spectrum. The inclusion of both early- and late-onset cases enhances clinical representativeness. Nonetheless, several limitations must be acknowledged. The modest sample size and inclusion of both MCI and AD stages limited statistical power for subgroup analyses, although this heterogeneity mirrors real-world patient diversity. Correlations between fluid biomarkers (CSF and p-tau217), and tau-PET should be interpreted cautiously due to batch variability in CSF measurements. The lack of a cognitively normal group in CSF analyses to anchor our relationships may also have influenced the detection of certain correlations. Moreover, there was a temporal mismatch between the acquisition of CSF/neuropsychological assessment and plasma biomarkers/tau-PET imaging, which may have introduced additional variability in the observed associations. Especially the differential associations of CSF and plasma p-tau species with tau pathology, should be interpreted cautiously. CSF p-tau was strongly associated with tau-PET despite the time gap between measurements, whereas CSF and plasma showed no association even when sampled concurrently. This pattern suggests that the CSF–plasma disconnect most probably reflects biological differences than timing. Prior studies indicate that CSF p-tau181 reflects broader intrathecal tau dysregulation and neuronal injury, while plasma p-tau217 more specifically tracks amyloid-related AD tau phosphorylation [55, 56]. Because CSF p-tau217 better detects amyloid pathology and aligns more closely with plasma p-tau217 [57], the lack of correlation between plasma p-tau217 and CSF p-tau181 in our cohort likely indicate that these markers capture related but biologically distinct aspects of AD pathophysiology. Although only one interaction term was performed, given the modest sample size and the number of brain regions and cognitive outcomes examined, the age group interaction analyses should be interpreted as exploratory and may be underpowered for some of the models. Replication in independent cohorts is warranted. Finally, the cross-sectional design precludes causal inference. Future longitudinal studies are needed to evaluate how PET and plasma p-tau217 differentially predict cognitive and structural decline over time. Furthermore, longitudinal data will determine whether plasma p-tau217 continues to increase linearly with tau burden or diverges at higher disease stages, and whether this relationship remains consistent across different assay platforms.

## Conclusions

While both plasma p-tau217 and tau-PET reflected disease stage, as indicated by cognitive and functional impairment, tau-PET additionally captured inter-individual heterogeneity in the spatial extent and burden of tau pathology. This was particularly evident in neocortical regions, where a marked phenotype-dependent asynchrony was observed: similar plasma p-tau217 levels reflected vastly different regional tau burdens depending on the age at onset. Therefore, tau-PET appears better suited for tracking disease severity and evaluating treatment effects, especially in those EOAD cases who exhibit higher tau-PET levels even in prodromal stages. Together, these results underscore the importance of tau-PET as a critical biomarker for assessing disease severity and support its central role in clinical trials aimed at evaluating disease-modifying therapies. Moreover, the distinct patterns of tau accumulation observed in early-onset versus late-onset AD highlight the need to account for age-related heterogeneity when designing clinical trials and precision treatment strategies. Integrating plasma, CSF, and tau-PET measures—while considering age-at-onset and cognitive phenotype—will be essential for refining diagnostic algorithms and improving stratification in disease-modifying treatment.

## Supplementary Information

Below is the link to the electronic supplementary material.

Supplementary file1 (PDF 13514 KB)

## Data Availability

The data that support the findings of this study are available from the corresponding author upon reasonable request. Due to participant privacy and ethical restrictions, individual-level imaging and clinical data cannot be made publicly available.

## Abbreviations

AD: Alzheimer’s disease
Aβ: Amyloid-β
PET: Positron emission tomography
pTau: Phosphorylated tau
GFAP: Glial fibrillary acidic protein
NFL: Neurofilament light chain
CSF: Cerebrospinal fluid
MRI: Magnetic resonance imaging
MCI: Mild cognitive impairment
EOAD: Early onset alzheimer’s disease
LOAD: Late onset alzheimer’s disease
SUVR: Standardized uptake value ratios
MNI: Montreal neurological Institute
TIV: Total intracranial volume
MMSE: Mini-mental state examination
RAVLT: Rey Auditory verbal learning test
RO: Rey-osterrieth complex figure
TMT: Trail making test
WAIS-IV: Wechsler adult intelligence scale – Fourth Edition
FWE: Family-wise error
GAMs: Generalized additive models
FDR: False discovery rate
