# Supplementary material for: [18F]RO948 Tau PET in early and late onset Alzheimer’s disease: associations with plasma p-tau 217, atrophy and cognition
Source: Eur J Nucl Med Mol Imaging. 2026 Jun 26;53(12):7359–75. doi: 10.1007/s00259-026-08015-w (PMC13633377; doi:10.1007/s00259-026-08015-w)
Supplement: Supplementary file 1 [file 259_2026_8015_MOESM1_ESM.pdf]

Variable	CN-young N = 8	CN-old N = 10	p.value
Plasma p-tau217	10.9 (0.411)	11.1 (0.369)	0.360
Entorhinal Cortex SUVR	1.26 (0.184)	1.21 (0.106)	0.829
Inferior Temporal Cortex SUVR	1.38 (0.206)	1.33 (0.0849)	0.965
Parietal Cortex SUVR	1.23 (0.132)	1.15 (0.104)	0.315

Table S1. Differences between CN-old and CN-young groups in selected variables.

**Variable**	**CN** N = 18	**MCI Aß-** N = 10	**MCI Aß+** N = 16	**AD** N = 13	**MCI-EOAD** N = 9	**EOAD** N = 5	**MCI-LOAD** N = 7	**LOAD** N = 8
Hippocampus SUVR	1.07 (0.07)	1.04 (0.08)	1.47 (0.21)[a]	1.57 (0.24)[a]	1.47 (0.26)	1.61 (0.23)	1.46 (0.14)	1.55 (0.25)
Amygdala SUVR	0.94 (0.08)	1.01 (0.11)	1.82 (0.49)[a]	2.17 (0.44)[a]	1.76 (0.54)	2.00 (0.45)	1.89 (0.47)	2.28 (0.42)
Frontal Cortex SUVR	1.13 (0.08)	1.10 (0.10)	1.23 (0.20)	1.55 (0.53)[a]	1.28 (0.24)	1.80 (0.63)	1.17 (0.14)	1.40 (0.42)
Occipital Cortex SUVR	1.30 (0.16)	1.26 (0.15)	1.58 (0.69)	1.71 (0.39)[ab]	1.78 (0.86)	1.95 (0.53)	1.31 (0.21)	1.57 (0.19)
Hippocampal Volume/TIV	2.65 (0.33)	2.08 (0.36)	2.30 (0.37)[a]	2.04 (0.31)[a]	2.40 (0.38)	2.06 (0.21)	2.17 (0.34)	2.03 (0.37)
Amygdala Volume/TIV	1.02 (0.16)	0.80 (0.15)	0.84 (0.16)[a]	0.72 (0.15)[a]	0.89 (0.19)	0.70 (0.13)	0.78 (0.09)	0.74 (0.17)

Table S2. Biomarker and atrophy data for the different study groups. Values are presented as Mean (SD). Shown are SUVR values in the selected brain regions, plasma biomarker levels, and volumetric ratios across cognitively normal (CN)**,** mild cognitive impairment amyloid-negative (MCI Aβ–)**,** mild cognitive impairment amyloid-positive (MCI Aβ+)**,** and Alzheimer's disease (AD) groups, for both early-onset (EOAD) and late-onset (LOAD) cases.Group comparisons displayed: CN vs MCI Aβ+ vs AD, MCI-EOAD vs MCI-LOAD, EOAD vs LOAD.

a = statistically different from CN, b = statistically different from MCI AB+.

c = statistically different from MCI-EOAD, d = statistically different from EOAD. Significance level $p<0.05$. **Abbreviations:** SUVR = Standardized Uptake Value Ratio; p-tau = phosphorylated tau;GFAP = glial fibrillary acidic protein;NFL = neurofilament light chain;TIV = total intracranial volume.

Region	Model	F (interaction)	p-value	Dev. explained (%)	Adj R^2
Frontal	Age-only adj.	3.03	0.0447	58.5	0.466
	Fully Adjusted	3.62	0.0331	61.1	0.457
Parietal	Age-only adj.	15.95	4.01e-05	73.5	0.706
	Fully Adjusted	12.44	8.61e-05	76.5	0.735
Occipital	Age-only adj.	10.22	0.000217	65.1	0.548
	Fully Adjusted	8.75	0.000363	66.4	0.541
Inferior Temporal	Age-only adj.	3.19	0.0772	61.1	0.488
	Fully Adjusted	3.37	0.0322	71.0	0.553
Middle Temporal	Age-only adj.	1.29	0.293	65.2	0.535
	Fully Adjusted	1.67	0.194	70.8	0.559
Superior Temporal	Age-only adj.	2.03	0.121	64.5	0.576
	Fully Adjusted	2.41	0.0842	70.3	0.618
Entorhinal	Age-only adj.	6.46	0.0304	71.2	0.605
	Fully Adjusted	1.97	0.150	72.8	0.625
Amygdala	Age-only adj.	17.46	0.00295	76.2	0.684
	Fully Adjusted	—	n.s.	82.6	0.740
Hippocampus	Age-only adj.	4.83	0.0435	64.7	0.590
	Fully Adjusted	2.56	0.0761	74.8	0.699

Table S3. Summary of Generalized Additive Models (GAM) for the interaction of plasma p-tau217 by age group across brain regions. The table provides statistical parameters for both unadjusted and adjusted models. Age-only adjusted models include the interaction between plasma p-tau217 and age group (EOAD vs. LOAD) as the primary smooth term with age as covariate. Fully adjusted models include sex and APOE4 status as additional covariates.

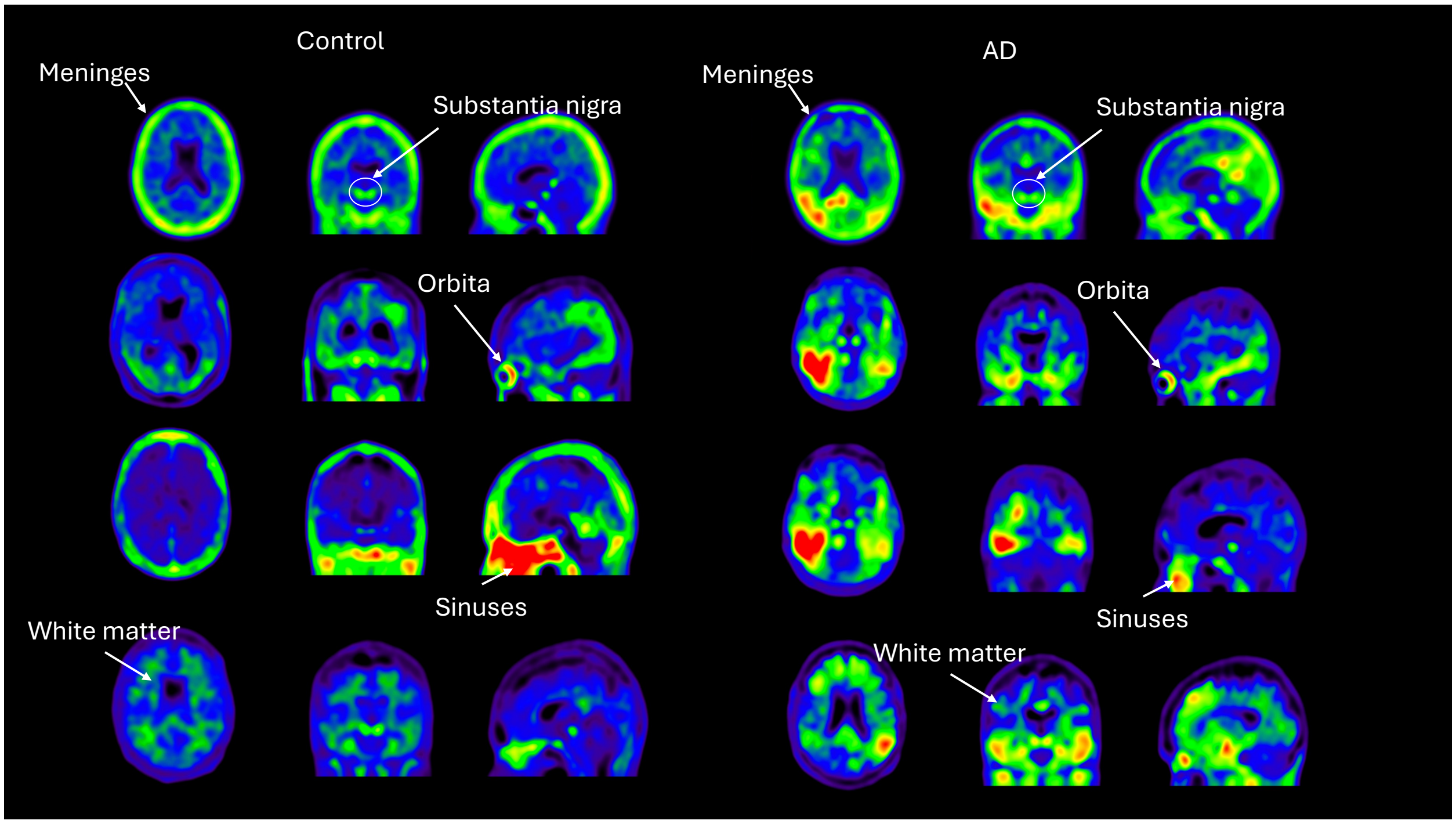

Fig. S1. Representative tau PET images across Control and AD groups representing off target binding.

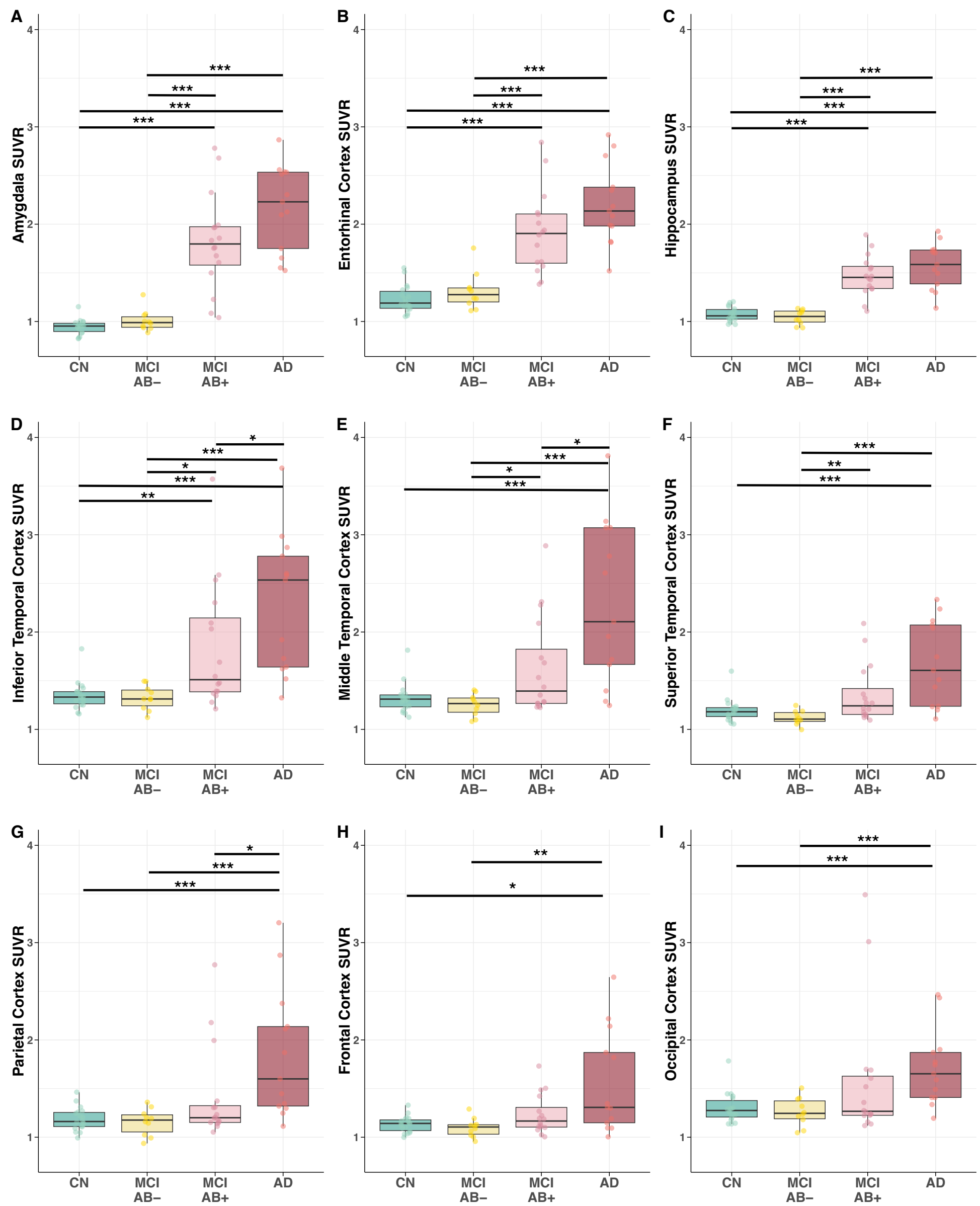

Fig. S2. Regional differences in [^{18}F]RO948 tau PET Standardized Uptake Value Ratio (SUVRs) across Alzheimer's disease diagnostic groups. Boxplots showing Standardized Uptake Value Ratio (SUVR) values in the selected regions across cognitive normal (CN), mild cognitive impairment amyloid-negative (MCI Aβ-), mild cognitive impairment amyloid-positive (MCI Aβ+) and Alzheimer's Disease (AD) groups.

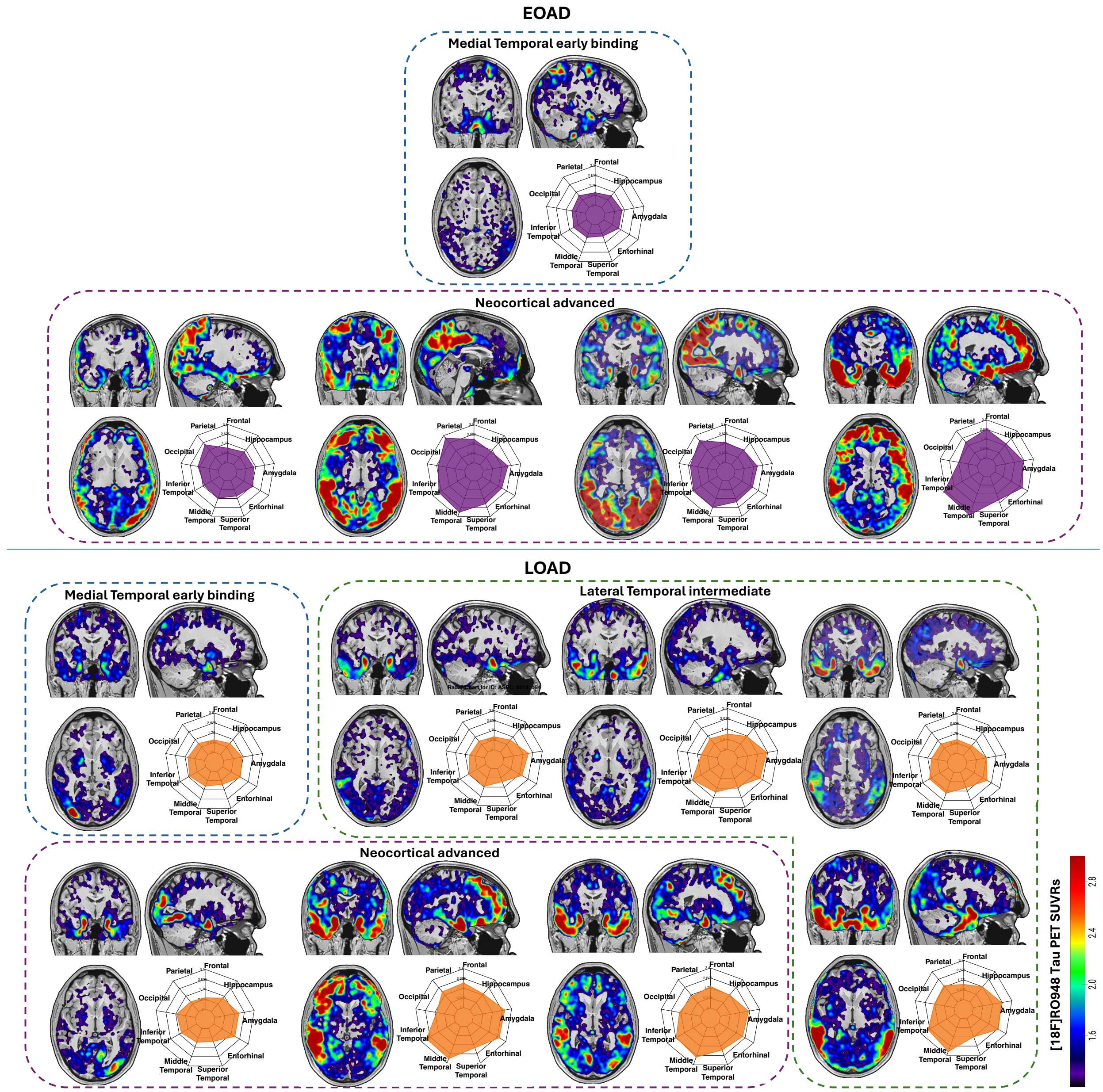

Fig. S3. [18F]RO-948 TAU binding distribution in AD groups by age-at onset. [^{18}F]RO948 tau PET scans (overlayed on corresponding MRI for anatomical reference) for Alzheimer's Disease (AD) individuals are shown and subdivided according to Early- (EOAD) and Late-Onset (LOAD) subgroups. For each subject, coronal, sagittal and axial views and radar plots display Standardized Uptake value Ratio (SUVR) values across key brain regions (Frontal, Parietal, Occipital, Inferior Temporal, Middle Temporal, Superior Temporal, Entorhinal, Amygdala, Hippocampus). Individuals were categorized according to the regional extent of [^{18}F]RO948 binding, reflecting progressive stages of tau accumulation from medial temporal to neocortical regions.

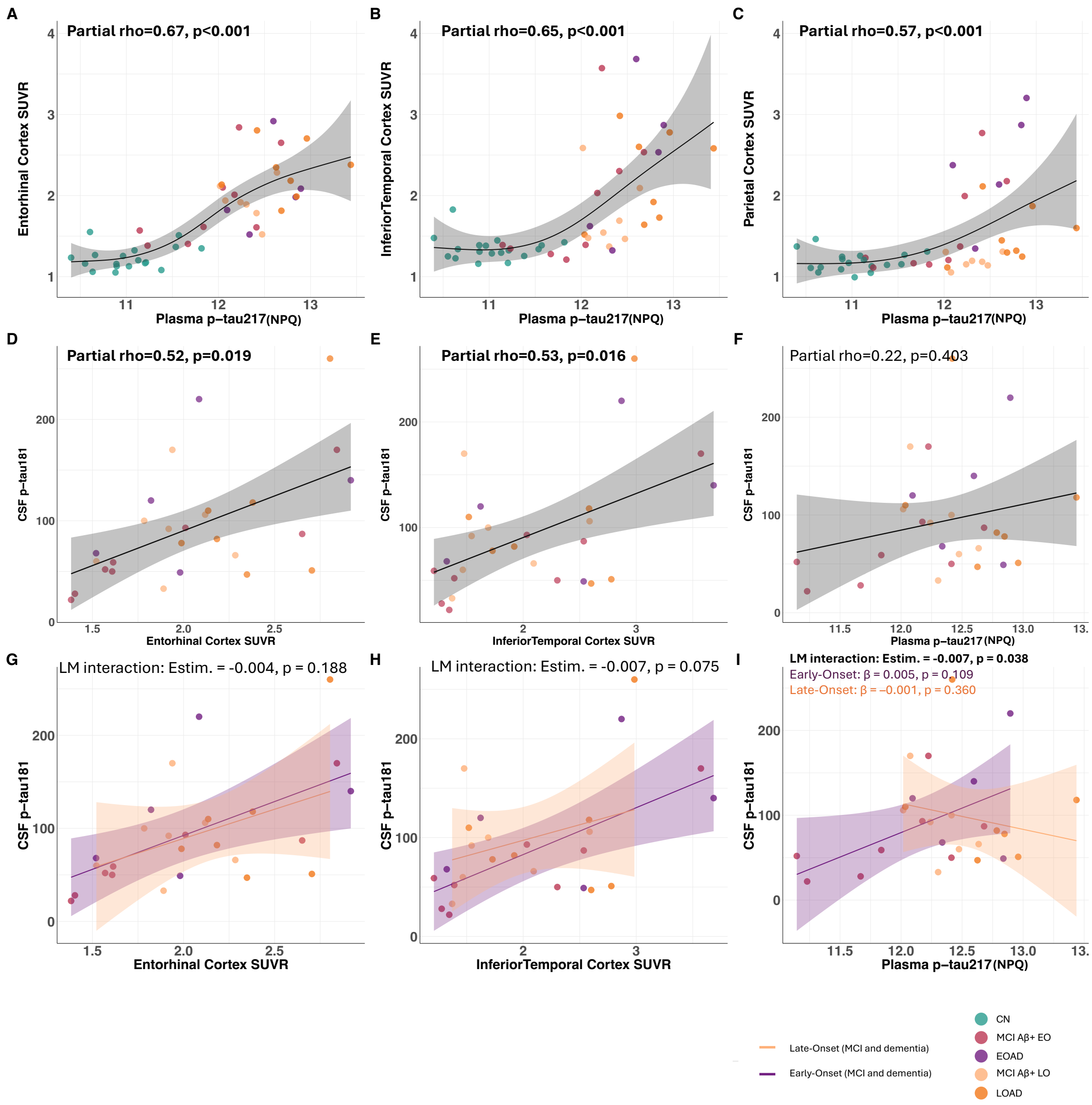

Figure S4. Regional correlations between [18F]RO948 tau-PET binding and plasma p-tau217 and their correlation with CSF p-tau181 across Alzheimer's disease diagnostic groups. Panels **(A-C)** display associations between tau-PET Standard Uptake Value Ratio (SUVR) and plasma phosphorylated tau (p-tau217) in main regions explored of **(A)** entorhinal, **(B)** inferior temporal, **(C)** and parietal cortices. Panels **(D-E)** display correlations between Cerebrospinal Fluid (CSF) phosphorylated tau (p-tau181) and tau-PET SUVR, **(F)** correlations between CSF p-tau181 and plasma p-tau217. **(G-I)** Associations between

CSF p-tau181 and regional tau PET SUVRs in the entorhinal **(G)**, inferior temporal cortex **(H)**, and p-tau217 **(I)**. Smooth curves were fitted using generalized additive models (GAMs) with a Gamma distribution and log link **(A-C)** or linear models (LMs)**(D-I)**. Shaded areas indicate 95% confidence intervals. Separate lines are fitted for early-onset AD (purple line) and late-onset AD (orange line) (G-I). Colored dots indicate individual data points across five groups: cognitively normal (CN), early-onset and late-onset mild cognitive impairment (MCI-EO/MCI-LO) or Alzheimer's Disease (EOAD/LOAD). Partial Spearman's rho and FDR-corrected p-values are reported within each panel (n=47). Partial correlation coefficients (rho) and p-values are derived from models controlling for Sex and APOE status. NULISA plasma p-tau217 levels are expressed in NULISA Protein Quantification (NPQ) units.

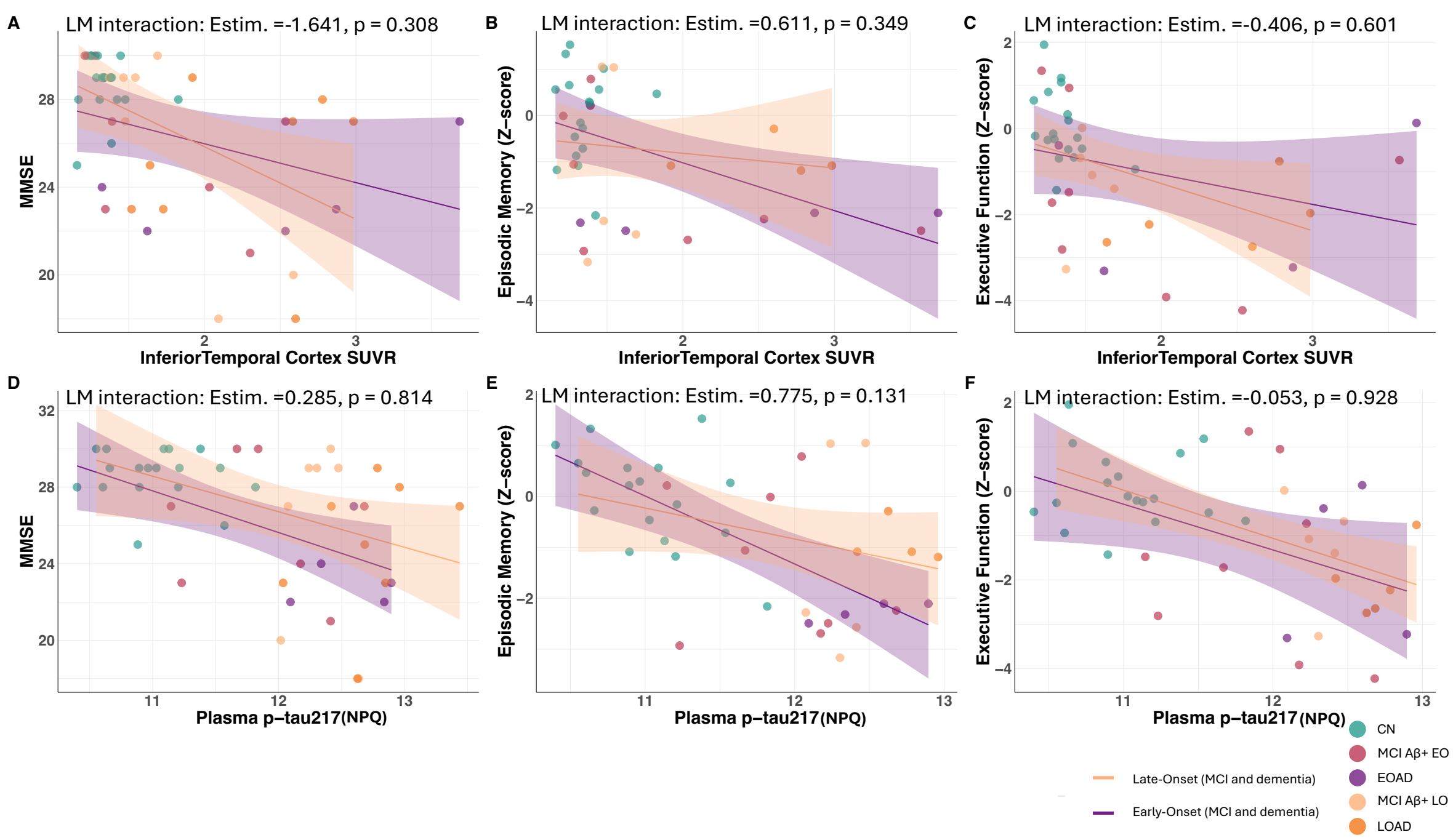

Fig. S5. Regional regressions between [^{18}F]RO948 tau PET binding, plasma biomarkers and cognition across Alzheimer's disease diagnostic groups. (Panels A-C) Regressions between Inferior Temporal [^{18}F]RO948 SUVRs and **(A)** MMSE **(B)** Episodic Memory **(C)** Executive Function (**Panels D-F)** Associations between plasma p-tau217 and **(D)** MMSE **(E)**Episodic Memory **(F)** Executive function MMSE(n=45), Episodic Memory z-score(n=39),and Executive Function Z-score (n=40).Scatter plots show raw data points and unadjusted regression lines by EOAD/LOAD. Shaded areas represent 95% confidence intervals. LM interaction estimates and p-values are derived from models controlling for Sex and APOE status.p-tau= phosphorylated tau, SUVR= Standardized Uptake Value Ratio, MMSE= Mini-Mental State Examination. **Group abbreviations:** CN = cognitively normal; MCI-EO/MCI-LO= early-onset and late-onset mild cognitive impairment; EOAD/LOAD = early-onset and late-onset Alzheimer's disease.

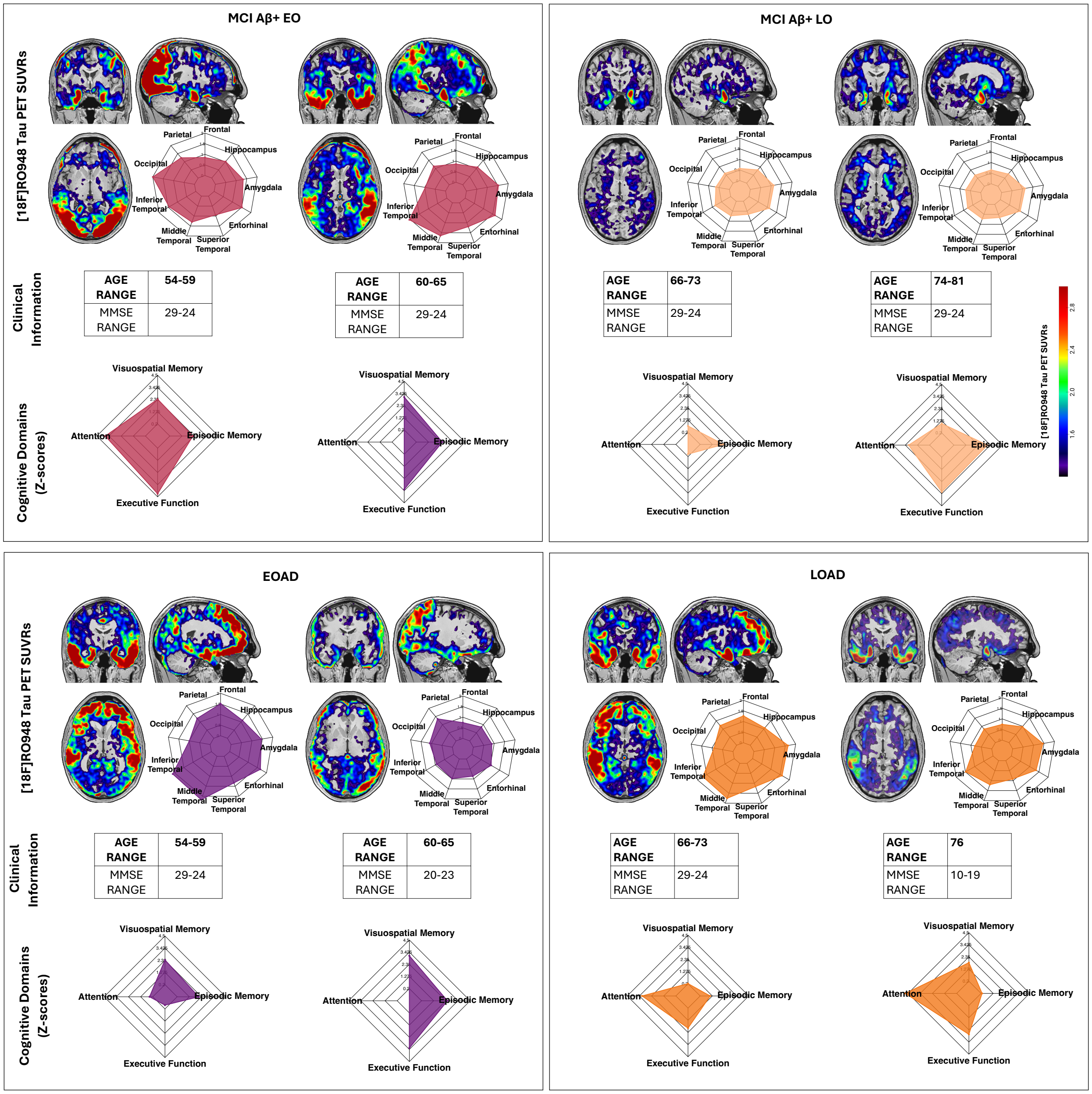

Fig. S6. Representative [18F]RO948 tau PET images, regional SUVR profiles, and cognitive/clinical features across Alzheimer's disease diagnostic groups. Representative [18F]RO948 tau-PET scans (overlayed on corresponding MRI for anatomical reference) are shown for two individuals per diagnostic group: mild cognitive impairment—early and late-onset (MCI-EO and MCI-LO), and Alzheimer's disease—early and late-onset (EOAD and LOAD). For each subject, radar plots display Standard Uptake Value Ratios (SUVR) values across key brain regions (Frontal, Parietal, Occipital, Inferior Temporal, Middle Temporal, Superior Temporal, Entorhinal, Amygdala, Hippocampus), along with corresponding radar plots of cognitive performance profiles. Individual-level clinical data including age, years of education, and MMSE scores are also shown. Regional tau uptake increases progressively from MCI-amyloid positive (Aβ+) toward dementia stages, with distinct neocortical involvement patterns

differentiating EOAD and LOAD. Radar plots emphasize group-specific topographies of tau burden and associated cognitive phenotypes. MMSE= Mini-Mental State Examination.

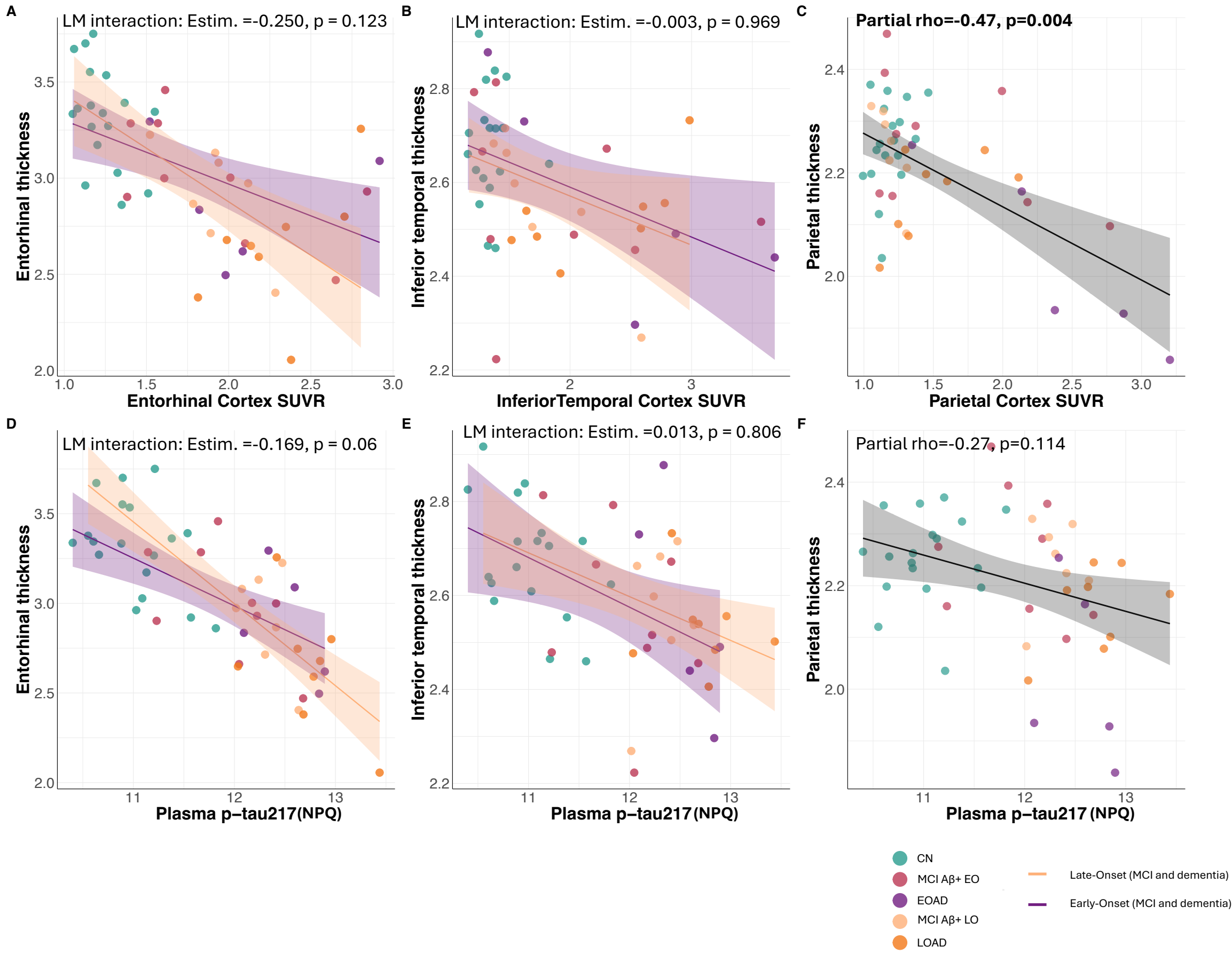

Fig. S7. Significant correlations between Tau PET or Plasma Biomarkers and Cortical Thickness. Panels A-C) Associations between **(A)** Entorhinal [18F]RO948 SUVR and Entorhinal thickness**(B)** Inferior Temporal [18F]RO948 SUVRs and Inferior Temporal thickness **(C)** Parietal [18F]RO948 SUVRs and Parietal thickness (**Panels D-F)** Associations between plasma p-tau217 and **(D)** Entorhinal thickness **(E)**Inferior Temporal thickness **(F)** Parietal thickness. Scatter plots show raw data points and unadjusted regression lines by EOAD/LOAD. Shaded areas represent 95% confidence intervals. LM interaction estimates and p-values are derived from models controlling for Sex and APOE status. Panel **(C,F)** fitted lines are presented for the entire sample and partial correlation coefficients (rho) and p-values are derived from models controlling for Sex and APOE status. SUVR= Standard Uptake Value Ratio. **Group abbreviations:** CN = cognitively normal; MCI-EO/MCI-LO= early-onset and late-onset mild cognitive impairment; EOAD/LOAD = early-onset and late-onset Alzheimer's disease.
